# 4D Printing of Magnetically Responsive Shape Memory Polymers: Toward Sustainable Solutions in Soft Robotics, Wearables, and Biomedical Devices

**DOI:** 10.1002/advs.202513091

**Published:** 2025-08-22

**Authors:** Kiandokht Mirasadi, Mohammad Amin Yousefi, Liuchao Jin, Davood Rahmatabadi, Majid Baniassadi, Wei‐Hsin Liao, Mahdi Bodaghi, Mostafa Baghani

**Affiliations:** ^1^ School of Mechanical Engineering College of Engineering University of Tehran Tehran 1417614411 Iran; ^2^ Department of Mechanical and Automation Engineering The Chinese University of Hong Kong Shatin, N.T. Hong Kong China; ^3^ Department of Engineering School of Science and Technology Nottingham Trent University Nottingham NG11 8NS UK

**Keywords:** 4D printing, Fe_3_O_4_, Magneto‐responsive SMPs, Multifunctional structures, Remote actuation, Smart materials

## Abstract

The fusion of 4D printing and magneto‐responsive shape memory polymers (SMPs) is unlocking new frontiers in remote actuation, reconfigurable materials, and multifunctional structures. This review provides a comprehensive analysis of the latest advancements in the fabrication, material selection, and application of these smart materials. The discussion encompasses the primary 3D printing techniques utilized for processing magneto‐responsive SMPs, including material extrusion, vat photopolymerization, and powder bed fusion. A critical comparison of fabrication methods highlights the influence of melt mixing and solvent casting on filler dispersion, mechanical performance, and actuation efficiency. Furthermore, various polymer matrices, such as thermoplastics and thermosets, are examined in conjunction with magnetic fillers, including Fe_3_O_4_, carbonyl iron powder (CIP), and neodymium magnet (NdFeB), to evaluate their effects on thermal, mechanical, and functional properties. The review also explores key application areas, such as biomedical engineering, soft robotics, and advanced wearable technology. Challenges related to material stability, actuation speed, and multi‐functional integration are discussed, along with emerging strategies to enhance performance and scalability. This work serves as a timely and in‐depth resource for researchers and engineers aiming to advance magnetic‐responsive materials in 4D printing toward sustainable soft robotic systems, biomedical devices, and flexible electronics.

## Graphical abstract

Merging 4D printing with magneto‐responsive shape memory polymers opens new avenues for intelligent, reconfigurable systems. This review navigates cutting‐edge fabrication techniques, magnetic fillers, and smart polymer matrices, unveiling their potential in soft robotics, biomedical devices, and wearable tech. Emphasis is placed on actuation performance, material design, and future scalability, offering a roadmap for next‐generation adaptive and sustainable applications.

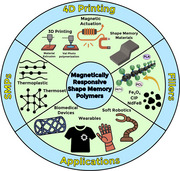

## 1 Introduction

The introduction of smart materials in printing technology has given rise to a groundbreaking concept known as 4D printing, where time is considered the fourth dimension.^[^ ^1^, ^2^ ^]^ 4D printing results from the rapid advancements and collaborative research in smart materials, 3D printers, and design. Compared to 3D printing, 4D printing adds the fourth dimension of time. This allows a printed structure to change its shape or function over time in response to external stimuli.^[^ ^3^, ^4^ ^]^ In addition to 3D printing, shape memory materials and actuation are the other two wings of 4D printing.^[^ ^2^ ^]^

SMPs are a type of smart material that has generated significant interest due to their ability to undergo 4D printing.^[^ ^5^, ^6^ ^]^ However, SMPs represent only one of several material systems capable of achieving shape transformation in 4D printed structures; other examples include hydrogels, liquid crystal elastomers, and composites with programmable behavior.^[^ ^7^ ^]^ These polymers can function as sensors and actuators, reacting to different stimuli, including direct and indirect thermal triggers (e.g., heat, magnetic fields, electrical currents), as well as other non‐thermal stimuli such as humidity, light, pH, and mechanical forces.^[^ ^8^, ^9^, ^10^, ^11^, ^12^, ^13^ ^]^ Moreover, as highlighted in recent studies,^[^ ^14^ ^]^ many conventional polymers, printed or non‐printed, exhibit intrinsic thermo‐ or chemo‐responsive shape memory effects, even without specialized modification.

The potential applications of SMPs span across aerospace, robotics, biotechnology, and tissue engineering. These applications utilize the sensing, actuation, self‐healing, and self‐diagnostic capabilities of SMPs,^[^ ^15^, ^16^, ^17^, ^18^ ^]^ and have inspired new directions in soft, sustainable, and biologically inspired robotics using biodegradable and bio‐based actuators.^[^ ^19^, ^20^ ^]^

Heat is the most common stimulus for triggering recovery through phase transitions in materials at different temperatures. While direct heating is typically used to activate shape memory effects (SME), it may not be appropriate for biomedical applications because of the potential risk of tissue damage.^[^ ^21^, ^22^ ^]^ In such instances, indirect heating is recommended. Magnetic‐sensitive SMP composites (MSMPC) have garnered significant interest from researchers across various fields, including medicine, because of their remarkable ability for remote activation.^[^ ^23^, ^24^ ^]^ Noncontact triggering of SME in polymers has been achieved by embedding magnetic nanoparticles such as iron oxide nanoparticles (Fe_3_O_4_) in thermally‐induced SMPs and inductively heating them in alternating magnetic fields,^[^ ^25^ ^]^ supporting advances in wearable magnetic sensing and interactive electronics,^[^ ^26^ ^]^ as well as enabling functionalities such as soft electromagnetic actuation^[^ ^27^ ^]^ and magnetically interactive electronic skin platforms for extended perception and augmented reality control.^[^ ^28^ ^]^

**Figure 1:**
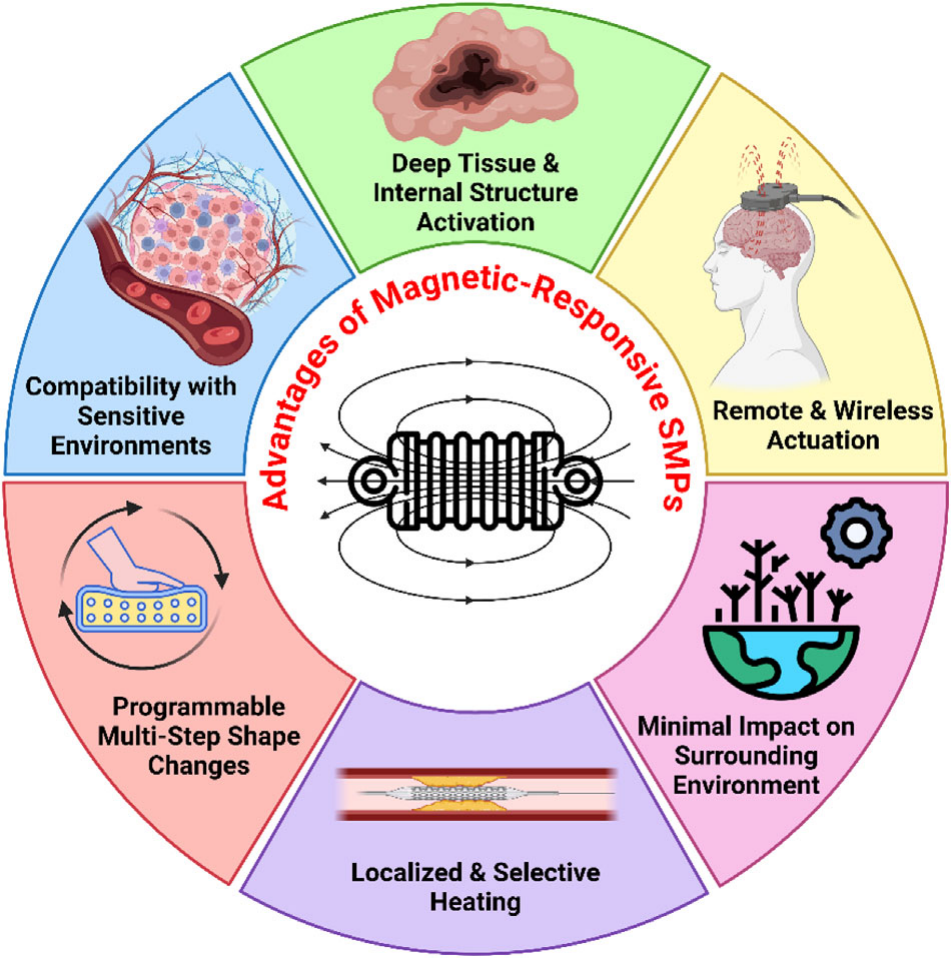
Advantages of magnetic‐responsive SMPs.

In this study, a review is conducted on the state‐of‐the‐art 3D printed magnetically activated SMP nanocomposites with 4D functionalities. To achieve this, a thorough exploration of the research on SMPs and their various types is first carried out, followed by an evaluation of studies in the field of 4D printing, with a particular focus on the use of the material extrusion and vat photopolymerization methods. Finally, the potential applications and challenges of magnetic SMP nanocomposites are discussed. **Figure** **1** illustrates a schematic representation of the key advantages offered by magnetically responsive composites, highlighting their unique capabilities over conventional SMPs.

## 2 4D Printing

4D printing integrates three core components: additive manufacturing (AM), shape memory materials, and external stimuli. First, 3D printing enables the precise fabrication of complex structures layer by layer. These structures are composed of shape memory materials, such as shape memory polymers or alloys, that can change their shape or function with time. The transformation is triggered by specific external stimuli, such as heat, light, moisture, pH, or magnetic fields. When exposed to the stimulus, the printed object responds by altering its geometry or properties, thereby adding the fourth dimension, time, to traditional 3D printing. This synergy enables the creation of smart, adaptive systems with potential applications across biomedical and soft robotics fields.

### 2.1 3D Printing

AM methods have been developed to meet the demand for high‐precision printing of complex structures. Rapid prototyping, the ability to produce large‐scale structures, minimizing printing defects, and enhancing mechanical properties are some of the key drivers behind the advancement of AM technologies. Among the seven main categories of 3D printing, material extrusion and photopolymerization are the most popular methods for making composites.^[^ ^29^ ^]^ In addition to these, emerging techniques such as Volumetric Additive Manufacturing (VAM) have also demonstrated potential for fabricating complex, stimuli‐responsive structures, including hydrogels with water‐responsive shape memory behavior.^[^ ^30^ ^]^

#### 2.1.1 Vat Photopolymerization

The Stereolithography (SLA) technique, developed in 1986, is an AM technique that uses a ultraviolet (UV)‐sensitive liquid polymer and a UV laser to construct structures layer by layer. When exposed to ultraviolet light, the polymer transitions into a solid form. Guided by computer software, the laser solidifies specific areas of each layer. After that, the platform lowers by a layer's thickness, usually between 50 and 150 µm. Fresh liquid polymer is spread across the surface, and the process is repeated until the 3D object is complete. After printing, any excess polymer is removed through a chemical bath, and the object undergoes UV curing for complete solidification and improved structural integrity.^[^ ^31^ ^]^ This post‐curing process not only enhances the mechanical strength and thermal resistance of the final part but also reduces internal stresses, leading to improved dimensional accuracy. SLA often uses acrylic or epoxy‐based monomers that polymerize quickly under UV light. Post‐processing, such as heating or additional curing, can be done to enhance mechanical performance.^[^ ^32^ ^]^ The mechanical behavior also strongly depends on the resin formulation; for instance, UV‐curable hybrid resins have shown excellent elasticity and body‐temperature‐responsive shape memory behavior.^[^ ^33^ ^]^

An advanced variant of SLA, called Digital Light Processing (DLP), uses a digital projector to cure entire surface layers at once, which enhances efficiency and scalability. Unlike SLA, where a laser traces each layer point by point, DLP projects a complete image of a layer, allowing faster fabrication times. This layer‐by‐layer projection results in shorter printing durations, making DLP particularly suitable for applications requiring rapid prototyping and high‐throughput production. DLP printers typically achieve similar resolutions to SLA (down to 25 µm), but with greater speed, making them ideal for manufacturing dental models, jewelry, and small mechanical components. Moreover, DLP systems often exhibit lower operating costs due to reduced energy consumption and faster cycle times.^[^ ^34^ ^]^ Both SLA and DLP offer high precision and surface finish quality compared to other 3D printing technologies; however, the choice between them depends on the specific balance required between speed, resolution, and material properties.

#### 2.1.2 Material Extrusion

**Figure 2:**
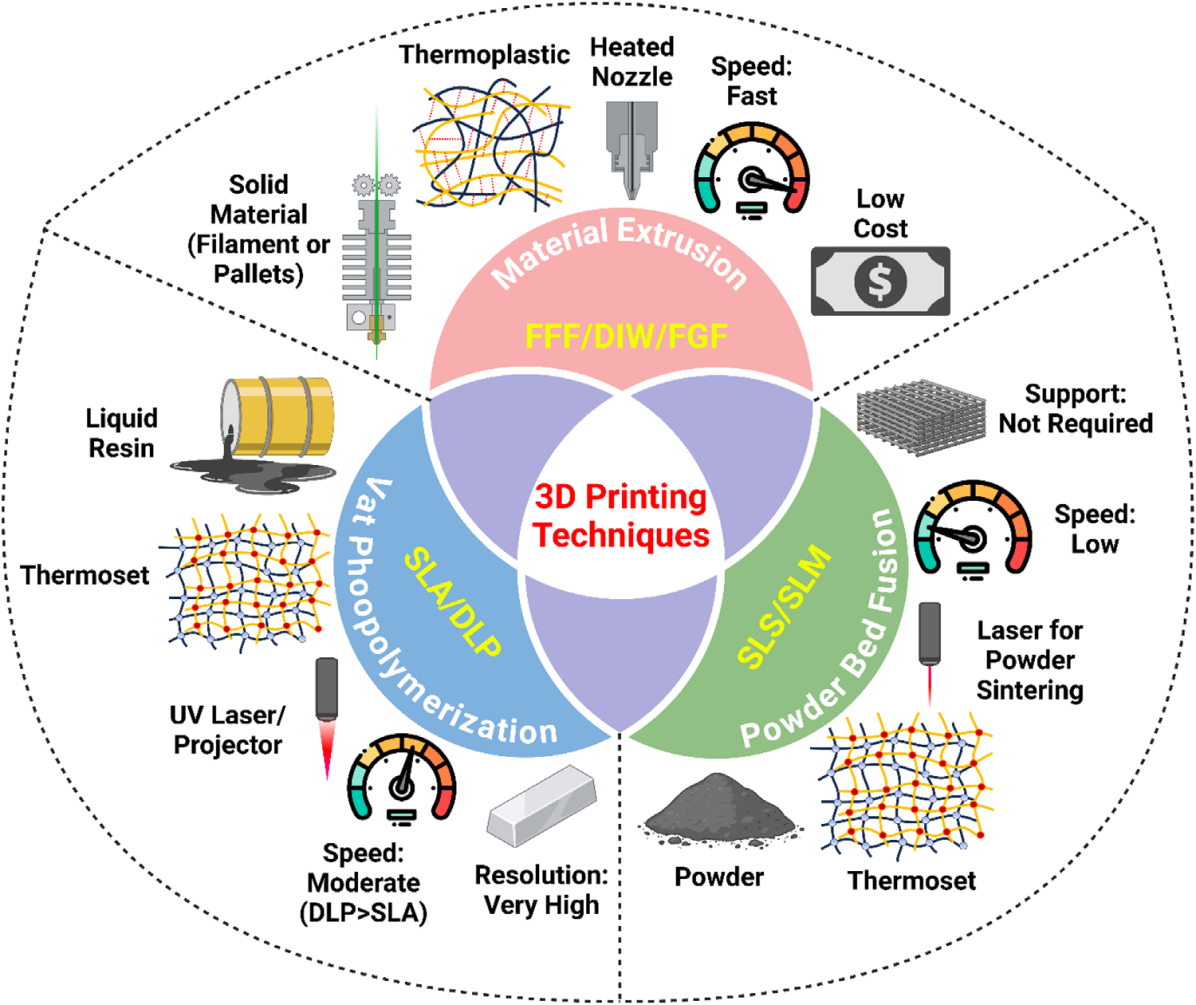
Comparison of material extrusion, vat photopolymerization, and powder bed fusion techniques for fabricating magnetic SMPs, highlighting their key properties, advantages, and limitations.

Materials extrusion is one of the most common methods in polymer‐based 3D printing,^[^ ^35^ ^]^ relying on the extrusion and deposition of material layers. In this process, materials are extruded through a printer nozzle using mechanisms such as pneumatic pressure, piston‐driven systems, or screw‐based feeding. The material is deposited layer by layer to create the desired structure. Materials extrusion comprises three primary techniques. Fused Deposition Modeling (FDM), Fused Granulate Fabrication (FGF), and Direct Ink Writing (DIW), each with distinct features. Several factors influence the printing precision and interlayer bonding strength in materials extrusion technologies. Material properties, especially thermal characteristics like melting point and thermal stability, significantly affect material flowability and cooling rates during printing. These factors are critical for achieving high accuracy and strong interlayer adhesion.^[^ ^36^ ^]^ **Figure** **2** highlights a comparison of three common 3D printing techniques used for fabricating magnetic SMPs: material extrusion, vat photopolymerization, and powder bed fusion (**Table** **1**).

**Table 1:** Key differences between vat photopolymerization (SLA/DLP) and material extrusion (FFF/DIW/FGF) 3D printing techniques.

Parameter	Vat Photopolymerization	Material Extrusion
Material State	Liquid resin	Solid filament/pellet
Polymer Type	Thermoset	Thermoplastic (can include thermoset via DIW)
Energy Source	UV laser/projector	Heated nozzle
Resolution	Very High	Moderate
Surface Finish	Smooth (best)	Rough (layered)
Support Structures	Required	Required
Mechanical Strength	Brittle to elastic, depends on resin (high detail)	Good but anisotropic
Post‐Processing	UV curing, cleaning	Minimal (sanding)
Print Speed	High (DLP > SLA)	Fast (low‐cost)
Applications	Jewelry, dental, models	Prototyping, functional parts
Cost	Moderate–High	Low

### 2.2 Shape Memory Polymers (SMPs)

SMPs, as a class of smart materials, display a diverse array of properties, ranging from stable to degradable, soft to hard, and elastic to rigid, all of which are influenced by their structural components.^[^ ^37^ ^]^ While most SMPs exhibit the ability to transition between two shapes (temporary and permanent), some specially engineered systems can exhibit multiple shape memory effects (multiple SME), recovering more than two shapes through distinct transitions or programming steps.^[^ ^14^, ^37^, ^38^ ^]^ Therefore, SMPs can actively transition from shape A to shape B. Shape A is a temporary shape obtained through mechanical deformation and the subsequent stabilization of that deformation. After this process, shape B, the permanent shape, is formed by applying the appropriate stimulus. This phenomenon is known as SME, which relies solely on molecular architecture and does not rely on specific chemical structures in the repeating units.^[^ ^39^ ^]^

SMPs are typically responsive to temperature.^[^ ^40^ ^]^ In the case of temperature‐sensitive SMPs, the thermomechanical cycle begins with the SMP in its permanent shape. When exposed to high temperatures, it undergoes mechanical loading and adopts a temporary shape. As the SMP is cooled below its transition temperature, the temporary shape becomes stable, a process known as programming. During the final stage, the material is reheated to restore its permanent shape. Two significant measures used to characterize the impact of shape memory are the shape recovery ratio (R_r_) and the shape fixity ratio (R_f_). The shape recovery ratio indicates a material's ability to return to its original shape, while the shape fixity ratio signifies its capacity to switch components to recover from mechanical deformation.^[^ ^41^ ^]^ A distinction can be made between two types of SMPs based on their behavior in cyclic processes: one‐way and two‐way. Notably, multiple SME can be achieved in both one‐way and two‐way SMPs, depending on their thermal programming and material design.^[^ ^5^, ^42^, ^43^ ^]^ An SMP is considered a two‐way SMP if, after the first thermomechanical cycle, it is cooled below its transition temperature and undergoes a transformation that allows it to return to its original shape. If the SMP fails to recover its temporary shape, it is known as a one‐way SMP, indicating that it can only function within a single thermomechanical cycle.^[^ ^44^, ^45^ ^]^

Based on chemical structure and processing behavior, SMPs are broadly classified into thermoplastics, thermosets, and, more recently, vitrimers. Thermoplastic SMPs offer recyclability and reprocessability,^[^ ^46^, ^47^ ^]^ while thermoset SMPs exhibit excellent mechanical strength, shape fixity, and thermal stability due to their permanent cross‐linked networks.^[^ ^48^, ^49^ ^]^ More recently, vitrimer‐like SMPs have emerged as a promising third category, combining dynamic covalent bonding with network integrity, enabling reshaping and healing capabilities, and are well‐suited for solid‐state UV cross‐linking and VAM.^[^ ^50^ ^]^

**Figure 3:**
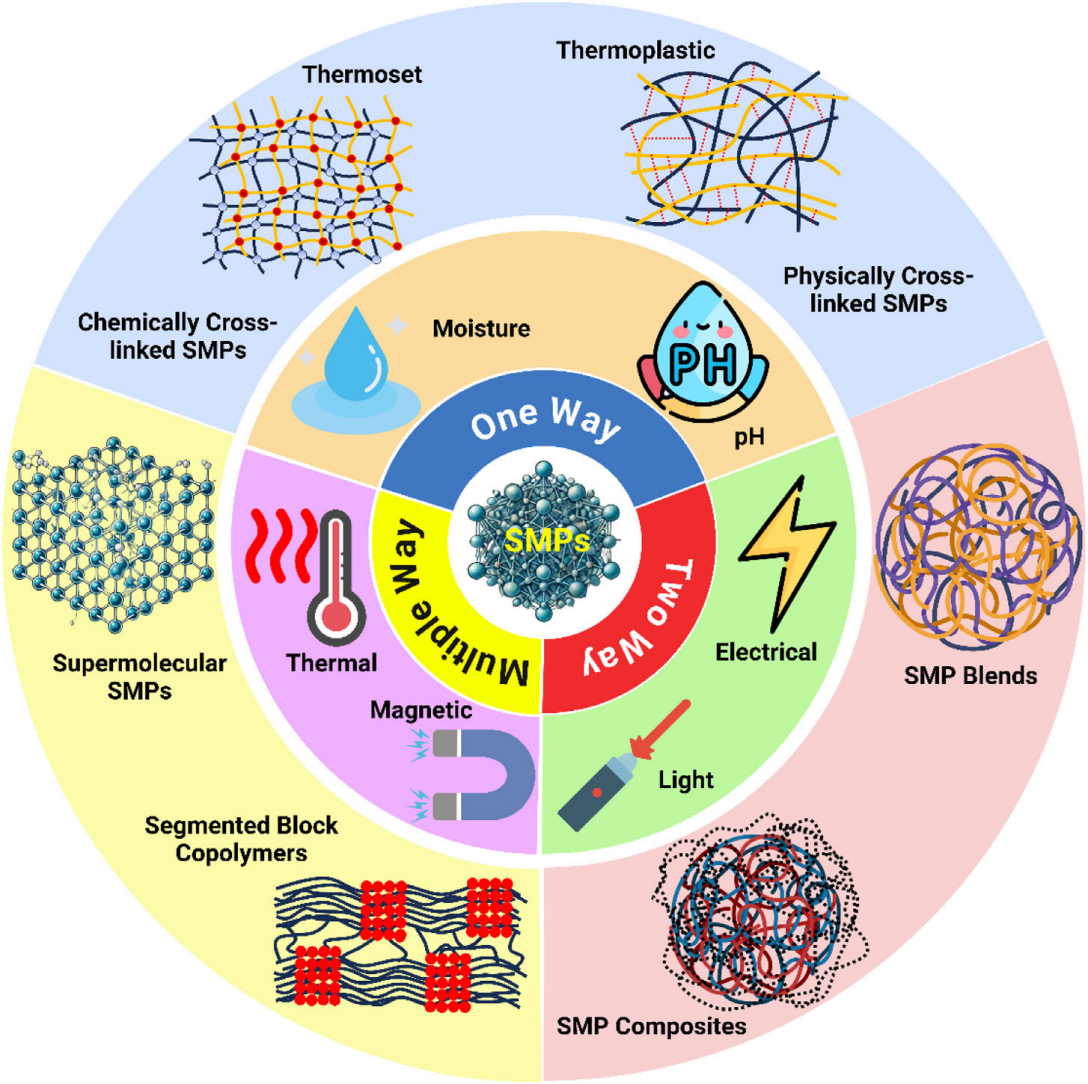
Classification of SMPs based on their structural composition, types of stimuli triggering shape recovery, and functional shape‐memory behaviors.

The classification of SMPs is typically based on three criteria: the composition and structure of the polymers, the type of stimulus that triggers the transition to the permanent phase, and the characteristics of shape memory performance.^[^ ^51^, ^52^ ^]^ **Figure** **3** provides a comprehensive overview of the classification of SMPs based on their structure, stimuli, and shape‐memory functionality.

### 2.3 Stimulus

#### 2.3.1 Thermal

Thermal‐responsive SMPs, comprising the most widely studied category, undergo shape recovery through temperature‐induced phase transitions. Two types of thermal response exist: heating‐responsive SME, which is intrinsic to most polymers, and cooling‐responsive SME, which occurs in specially designed systems such as hydrogels. Heat initiates this transition either directly, by raising the polymer temperature above its glass transition temperature (T_g_) or melting temperature (T_m_), or indirectly, via mechanisms such as Joule heating, photothermal conversion, or magnetic induction.^[^ ^53^, ^54^ ^]^ Cooling‐responsive SMPs, on the other hand, recover their original shape upon cooling below a certain threshold due to reversible phase transitions, such as sol–gel transformations in water‐content‐dependent hydrogels.^[^ ^30^ ^]^

##### Direct Actuation

Direct thermal stimulation can be achieved by increasing the ambient temperature using a warm environment, such as hot water or hot gas. This category encompasses the most prevalent SMPs. Amorphous polymers in this category regain their original shape when heated above their T_g_, while crystalline polymers regain their shape when heated above their T_m_. When the polymer exceeds the T_g_. The polymer chains start to vibrate and move, leading to the release of stored energy in the system, allowing the SMP to return to its initial state. Consequently, when the polymer deforms, there is a loss of entropy associated with elastic entropy. In the rubbery state, the polymer tends to recover its original shape by restoring the lost entropy.^[^ ^55^ ^]^ The activation process typically involves heating the SMP, deforming it under load, cooling to fix the temporary shape, and reheating to induce shape recovery.

##### Indirect Actuation

In indirect thermal actuation, the polymer is not heated directly but instead responds to external stimuli that produce localized heating. These include magnetic, electric, and photothermal methods.

*Magnetic*: Magnetic field‐sensitive SMPs can be created by preparing nanocomposites loaded with specific ferromagnetic nanoparticles, such as Fe_2_O_3_, Fe_3_O_4_, Ni, etc.^[^ ^11^, ^56^, ^57^ ^]^ The shape is recovered in a similar manner to how thermally inductive shape memory composites (SMPCs) regain their shape. Shape recovery is remotely demonstrated through the Joule effect when the sample is subjected to an external magnetic field. Ferromagnetic particles act as inductive heaters when subjected to an alternating magnetic field. This is achieved through mechanisms such as hysteresis loss, eddy current loss, and other methods that differ depending on the types and sizes of the magnetic particles.^[^ ^23^, ^58^, ^59^ ^]^

*Electric*: SME is observed when an electric current or voltage is applied. Shape memory characteristics can be generated in electrically responsive SMPs through Joule heating.^[^ ^8^, ^60^, ^61^ ^]^ Therefore, due to the insulating nature of the polymers, conductive fillers such as metal‐based particles, carbon black, carbon nanotubes, and graphene can be incorporated into the polymer matrix. This allows electric current to pass through the network of conductive fillers in the SMP. The internal temperature of the SMP increases through the phenomenon of Joule heating. When the temperature exceeds the transition temperature, it activates the SMP.^[^ ^9^, ^62^ ^]^

*Photothermal (Light)*: Light‐responsive SMPs incorporate photothermal agents (e.g., gold nanorods, graphene oxide) that convert absorbed light, particularly near‐infrared (NIR) radiation, into heat, activating the SME. When exposed to specific wavelengths of light, the polymers form light‐responsive cross‐links, enabling them to assume a new shape and maintain it even after the stress is removed. Subsequent exposure to a specific wavelength of light causes the cross‐links to break, allowing the polymers to return to their original shape. Light has an advantage over direct heating because optical activation does not pose the risk of tissue damage associated with thermal operations.^[^ ^61^, ^63^, ^64^ ^]^

#### 2.3.2 Nonthermal

Nonthermal stimulus‐responsive SMPs activate through environmental or chemical cues without relying on heat. In pH‐responsive systems, polymers containing ionizable groups such as carboxyl or amine moieties undergo structural changes when exposed to varying acidity or alkalinity, leading to shape recovery through swelling or contraction.^[^ ^65^ ^]^ Moisture‐responsive SMPs, often based on hydrophilic networks like polyethylene glycol (PEG) or poly(N‐isopropylacrylamide) (PNIPAM), absorb water and experience volumetric changes that trigger the shape memory effect.^[^ ^66^ ^]^ In chemically responsive SMPs, shape recovery is initiated by specific chemical reactions that alter the polymer network, such as bond cleavage or crosslinking modifications, enabling controlled actuation in targeted chemical environments.^[^ ^67^ ^]^

## 3 Magneto‐Responsive SMPs in 4D Printing

Interest in advanced polymeric nanocomposites has surged in recent decades. Incorporating nanoparticles into polymer matrices enhances mechanical properties and introduces novel functionalities. Typically, nanocomposites are created by combining a nanofiller with a polymer matrix. This process leads to enhanced mechanical strength, thermal stability, or thermo‐mechanical performance depending on the specific nanofiller utilized.^[^ ^68^ ^]^ Additionally, nanoparticles can provide unique functionalities, such as electrical conductivity,^[^ ^69^ ^]^ magnetic properties,^[^ ^70^ ^]^ antioxidant capabilities,^[^ ^71^ ^]^ and antimicrobial behavior.^[^ ^72^ ^]^

Magnetic particles, typically in nanometer or micrometer sizes, are integrated into the SMP matrix to facilitate quick and remote heating of the material when exposed to an electromagnetic field. Acting as internal micro‐antennas, the particles convert electromagnetic energy into Joule heat through magnetic field‐induced inductive heating.^[^ ^73^ ^]^ This innovative approach offers several notable advantages for SMP shape recovery. One of the key benefits is the ability to heat the material remotely, eliminating the need for direct physical contact or localized heating devices. Additionally, the heating rate can be significantly accelerated, resulting in faster actuation of the SMP. Moreover, integrating magnetic particles into SMPs creates new opportunities in medical applications.^[^ ^74^ ^]^ These particles allow for non‐invasive imaging techniques, such as fluoroscopy or computed tomography (CT) scans, to detect and monitor implanted devices without requiring additional surgical interventions for positioning or functionality assessments.

### 3.1 Preparation Methods

Melt mixing is a widely used and efficient technique for incorporating magnetic fillers into polymer matrices. In this method, magnetic fillers are added to the molten polymer matrix, where they are uniformly distributed using a compounding machine or internal mixer. The high temperature required to melt the polymer ensures that the magnetic fillers are effectively dispersed, resulting in homogeneous mixtures. Melt mixing is favored due to its simplicity, low cost, high speed, and the lack of need for chemical solvents. This method is especially useful for large‐scale industrial applications and is commonly used in the production of polymer nanocomposites. Additionally, studies have shown that melt mixing can maintain the structural integrity of the magnetic fillers while enhancing the mechanical properties and thermal stability of the resulting composite materials.^[^ ^75^, ^76^ ^]^

Solvent casting is another effective technique, especially when a higher degree of precision is required for the dispersion of magnetic nanoparticles in the polymer matrix. In this method, magnetic fillers are initially dissolved in suitable solvents, followed by dissolving the polymer matrix in the same solvent. The mixture is thoroughly blended to ensure a fine dispersion of the nanoparticles within the matrix. Afterward, the solvent is evaporated, leaving behind the final film or composite material. Solvent casting is ideal when the polymer is difficult to process at its melting temperature or when precise control over filler dispersion is required at the nanoscale to achieve superior performance. For example, when incorporating Fe_3_O_4_ into a polymer matrix, the solvent‐based approach allows for precise control over nanoparticle size, distribution, and surface interactions, which ultimately influence the magnetic properties and performance of the composite material. Furthermore, solvent casting can improve the surface characteristics of the fillers, such as wettability and adhesion to the polymer matrix, leading to better compatibility and enhanced properties of the final composite. This method is commonly used in the production of films and coatings where uniform distribution of magnetic nanoparticles is critical for optimizing magnetic behavior, such as in magnetic sensors or drug delivery systems.^[^ ^75^, ^76^, ^77^ ^]^

**Figure 4:**
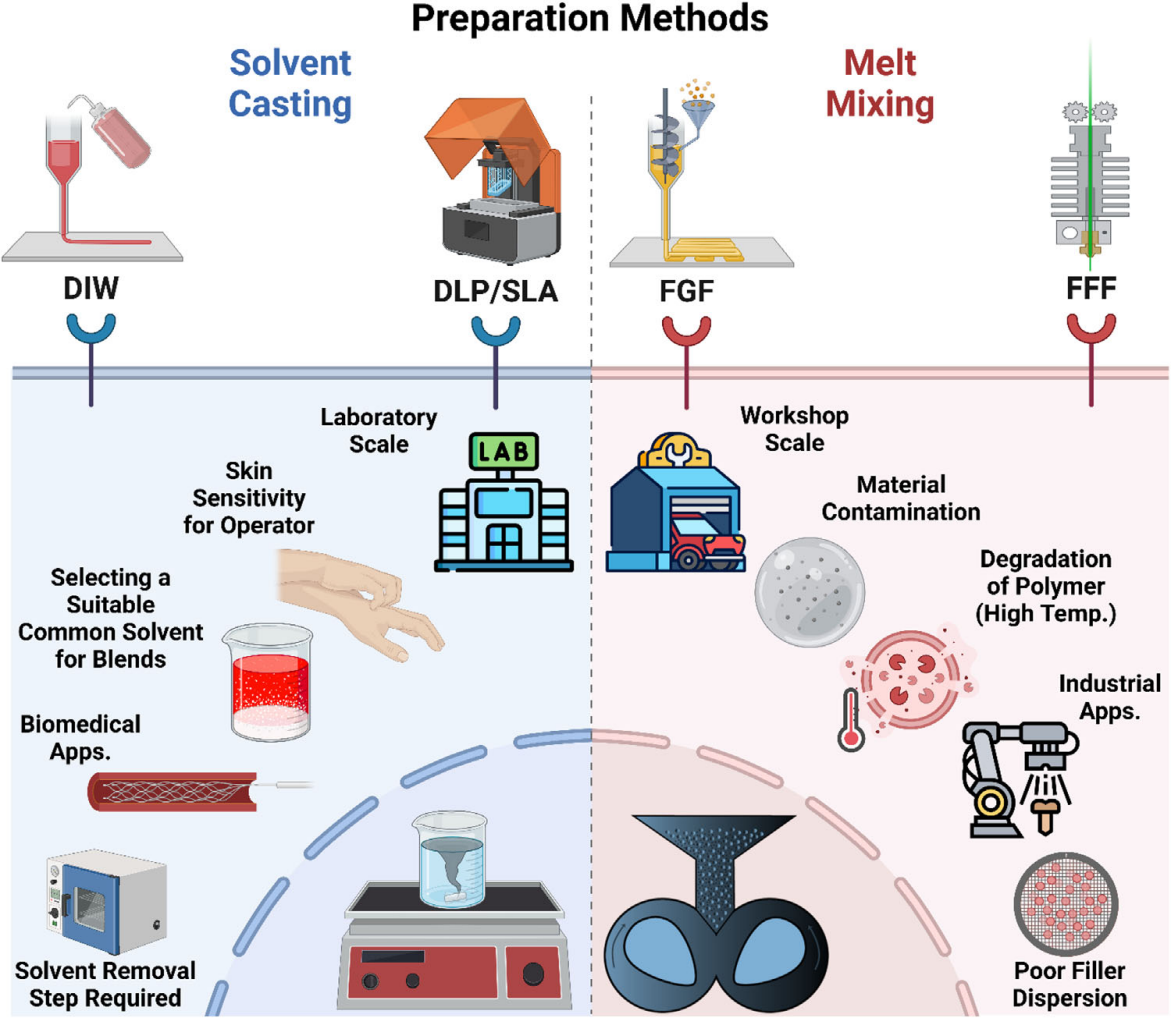
Comparison of melt mixing and solvent casting: key limitations in nanoparticle dispersion, processing, and material compatibility.

The choice between melt mixing and solvent casting depends on factors such as the nature of the polymer, the desired filler dispersion, processing conditions, and environmental considerations. For example, melt mixing is preferred for thermally stable polymers and when rapid processing is essential. On the other hand, solvent casting is suitable for polymers sensitive to high temperatures or when high filler dispersion is required. While both methods enable the fabrication of functional magnetic SMP composites, their limitations must be considered. Melt mixing often leads to increased material viscosity, making it challenging to process highly filled nanocomposites, whereas solvent casting requires careful solvent selection and extended drying times to prevent residual solvent contamination. Recent studies have explored hybrid methods that combine elements of both approaches to optimize filler dispersion and composite properties. **Figure** **4** illustrates a comparative overview of the disadvantages associated with each method.

### 3.2 Mechanism

Magnetic nanoparticles enhance the versatility of the SMP matrix by giving it magnetic properties. These nanoparticles can absorb and convert energy from an alternating magnetic field (AMF) into heat. When the SMP is exposed to an AMF, heat is generated due to hysteresis loss and eddy current loss. This inductive heat increases the material's temperature above its transition temperature, triggering its shape recovery process. Consequently, the magnetically actuated SMP transitions from its temporary shape back to its original form efficiently and reliably.^[^ ^70^ ^]^

### 3.3 Matrix

Matrix composites, especially those designed for 4D printing using magnetic field activation, come in various forms. This article analyzes the most common types, focusing on their mechanical behaviors, unique properties, benefits, and limitations. The discussion highlights their adaptability and practical applications in advanced manufacturing.

#### 3.3.1 Thermoplastics

##### PLA

**Figure 5:**
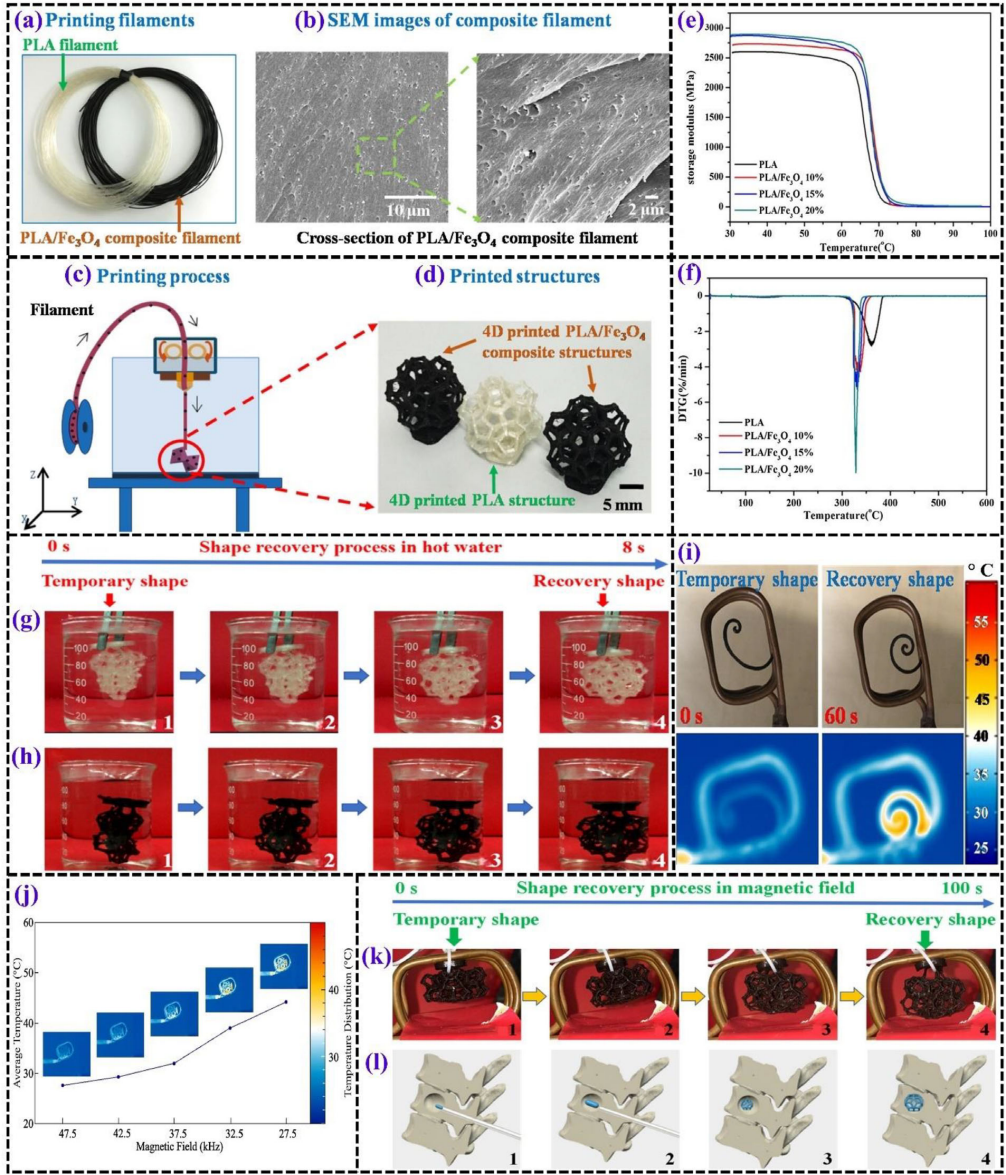
Overview of the 4D printing process and characterization of PLA and PLA/Fe_3_O_4_ composites. a) PLA and PLA/Fe_3_O_4_ filaments used for printing. Reproduced with permission.^[^ ^81^ ^]^ Copyright 2022, Elsevier Ltd. b) SEM images illustrating the composite filament morphology. Reproduced with permission.^[^ ^82^ ^]^ Copyright 2024, Elsevier Ltd. c) step‐by‐step depiction of the printing process, and d) final printed structures Reproduced with permission.^[^ ^81^ ^]^ Copyright 2022, Elsevier Ltd. Evaluation of PLA/Fe_3_O_4_ filaments with varying Fe_3_O_4_ content: e) DMA results and f) DTG analysis. Reproduced with permission.^[^ ^83^ ^]^ Copyright 2019, Elsevier Ltd. Thermal expansion responses of 4D‐printed complex structures in hot water: g) PLA and h) PLA/Fe_3_O_4_. Reproduced with permission.^[^ ^83^ ^]^ Copyright 2019, Elsevier Ltd. i) Magnetic field‐induced shape recovery of 4D‐printed structures containing 15% Fe_3_O_4_ at 27.5 kHz, including real‐time observations and thermal distribution. Reproduced with permission.^[^ ^84^ ^]^ Copyright 2021, Wiley‐VCH. j) Correlation between the average surface temperature and applied frequency under a magnetic field. Reproduced with permission.^[^ ^83^ ^]^ Copyright 2019, Elsevier Ltd. k) Shape recovery performance of the composite structure within a magnetic field. Reproduced with permission.^[^ ^81^ ^]^ Copyright 2022, Elsevier Ltd. (l) A simulation demonstrating the functionality of the 4D‐printed structure as a potential bone repair scaffold.Reproduced with permission.^[^ ^81^ ^]^ Copyright 2022, Elsevier Ltd.

**Figure 6:**
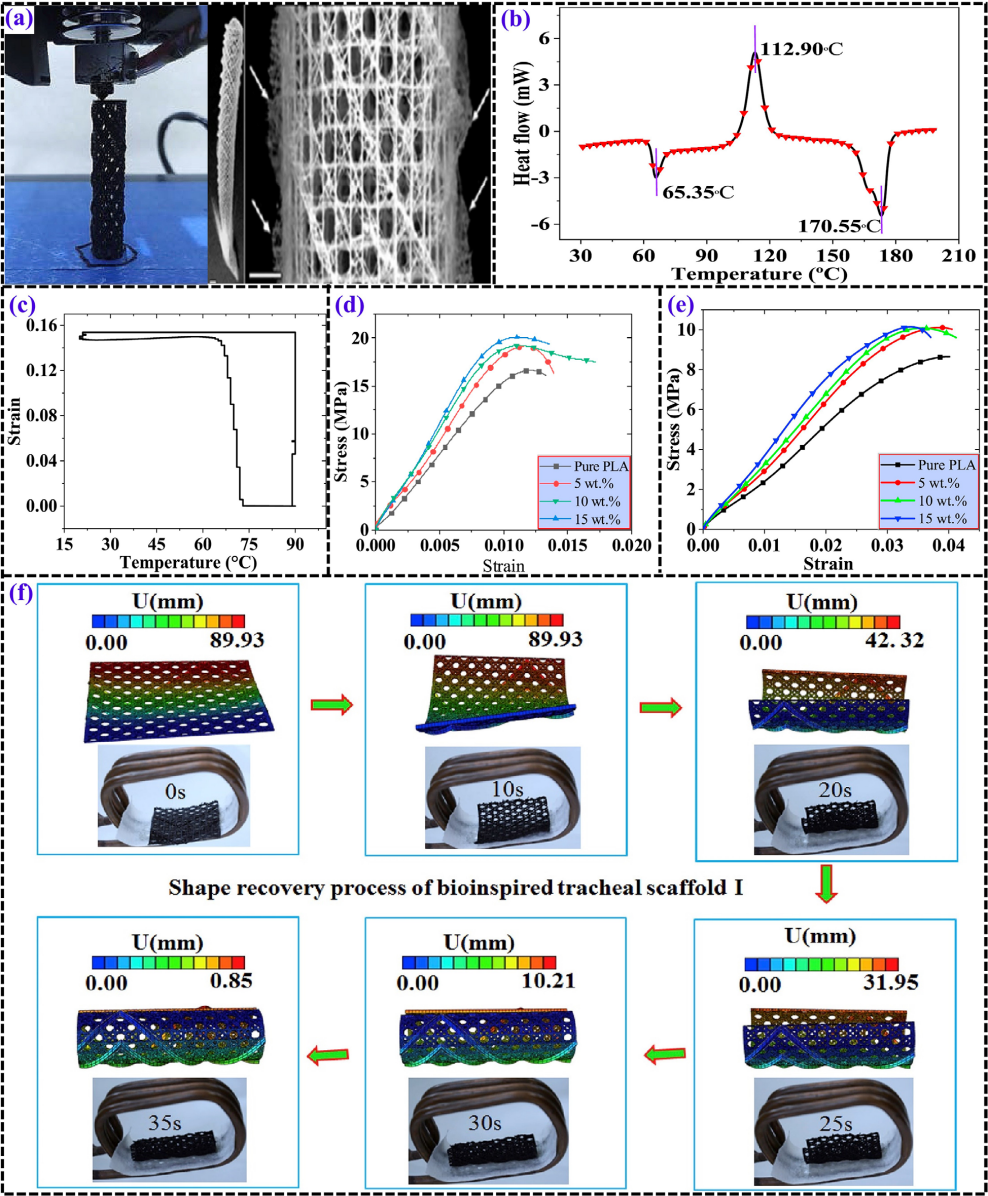
a) Bioinspired tracheal scaffolds fabricated using 4D printing, along with photograph showing the complete skeleton of a glass sponge alongside a fragment of its cage‐like structure. Reproduced with permission.^[^ ^85^ ^]^ Copyright 2021, Springer Nature. b) Differential scanning calorimetry (DSC) analysis showing thermal transitions. Reproduced with permission.^[^ ^86^ ^]^ Copyright 2019, Elsevier Ltd. c)Strain‐temperature relationship during the shape memory cycle. Reproduced with permission.^[^ ^86^ ^]^ Copyright 2019, Elsevier Ltd. Tensile performance of samples with varying particle concentrations at a test temperature of: d) 25°C (Room temperature), e) 37°C (Human body temperature). Reproduced with permission.^[^ ^86^ ^]^ Copyright 2019, Elsevier Ltd. f) Functional validation of bioinspired tracheal scaffold I, activated by a magnetic field in an in vitro environment. Reproduced with permission.^[^ ^85^ ^]^ Copyright 2021, Springer Nature.

**Figure 7:**
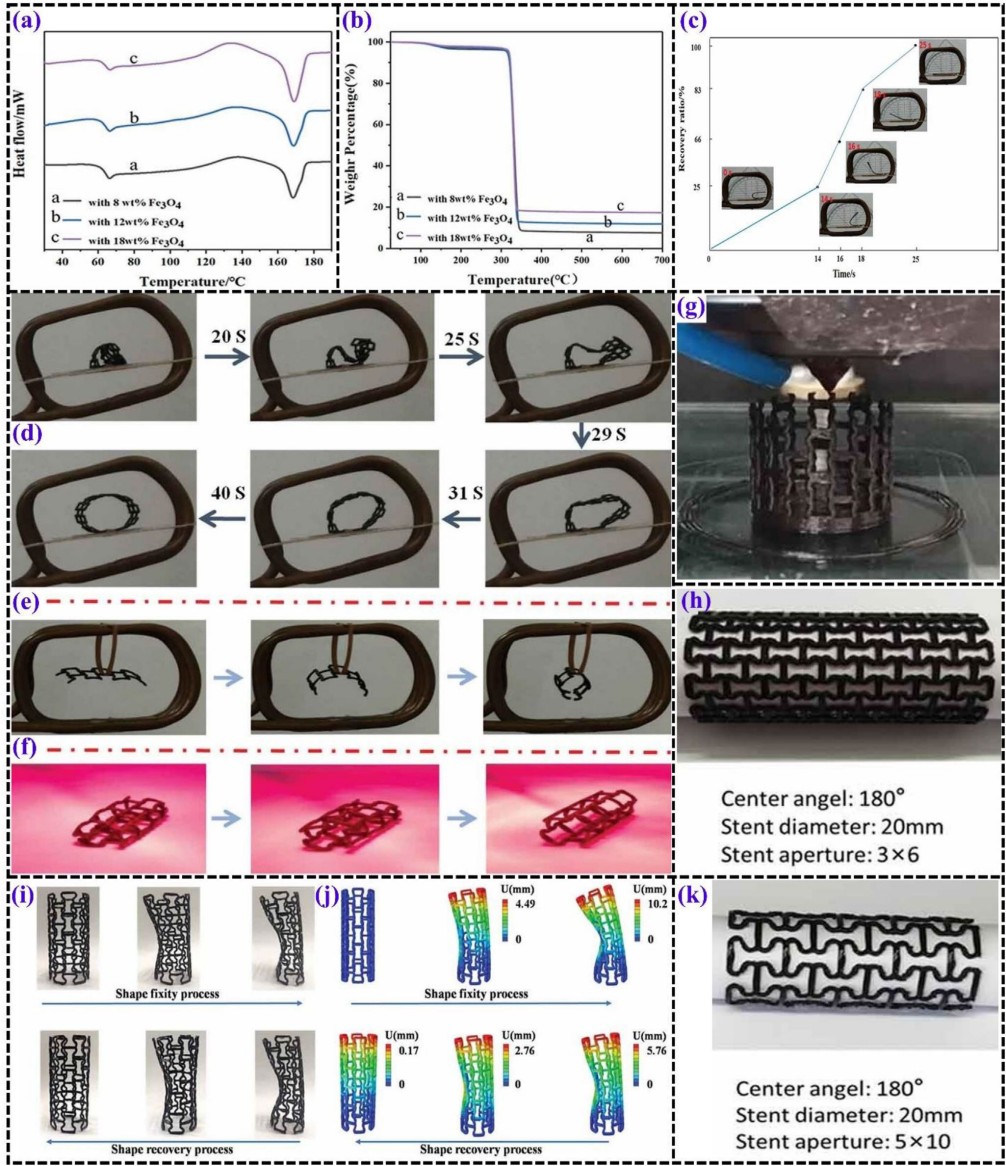
a) DSC and b) TGA curves of PLA/Fe_3_O_4_ SMPCs with varying Fe_3_O_4_ concentrations. c) Magnetic field‐induced shape recovery of a 2D SMP film fabricated via FDM. Recovery behavior of 4D‐printed PLA/Fe_3_O_4_ composite tracheal stents: d) stent dimensions of 2 × 4 mm, e) stent dimensions of 3 × 6 mm, and f) infrared light‐triggered shape recovery of the tracheal stent. g) Overview of the printing process. h) Printed sample with dimensions of 3 × 6 mm. Shape recovery of a tracheal stent bent at 30°: i) sequential images capturing the recovery process and j) simulation results. k) Another printed sample with dimensions of 5 × 10 mm.^[^ ^87^ ^]^

**Figure 8:**
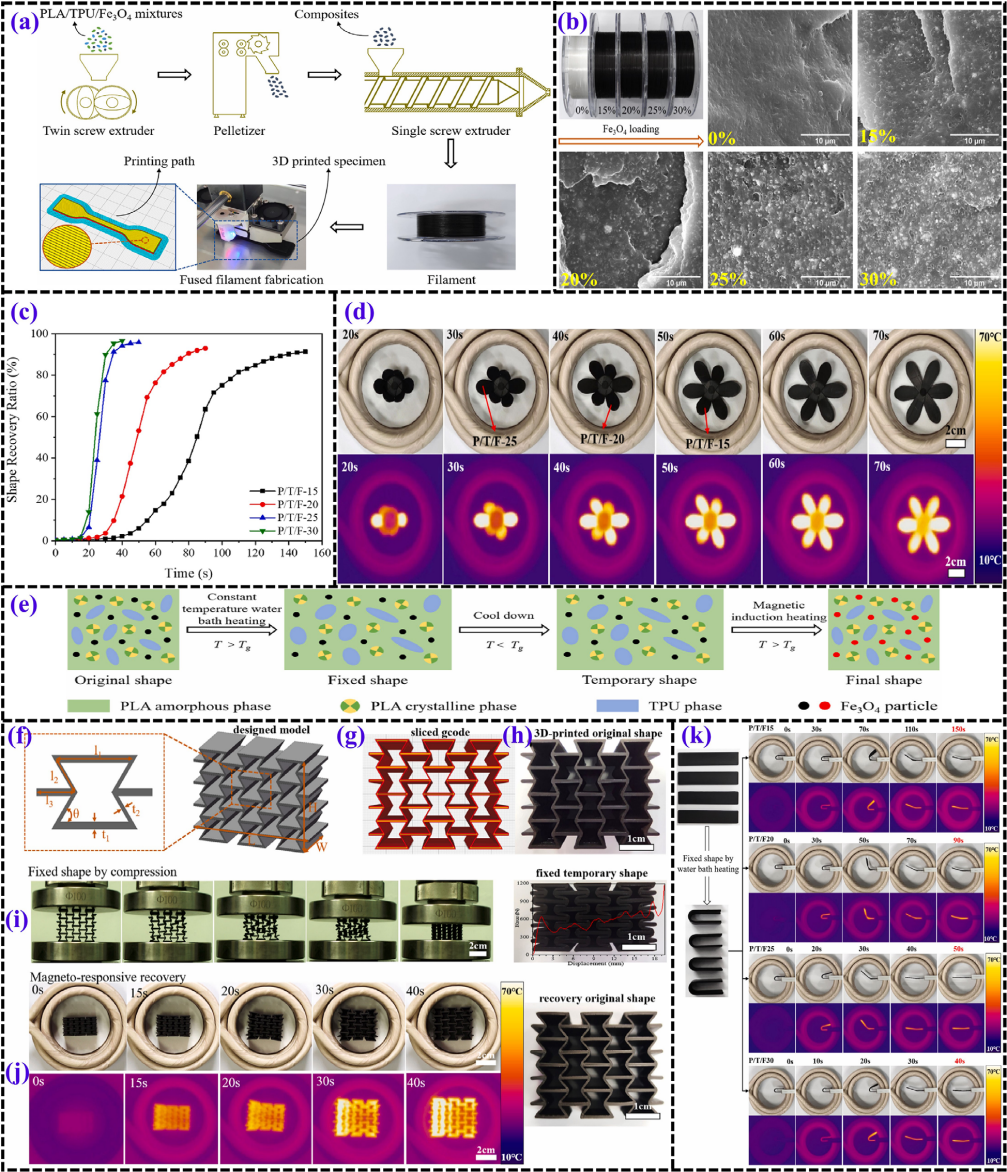
a) Step‐by‐step fabrication of 3D‐printed composite filaments and final products. Reproduced with permission.^[^ ^90^ ^]^ Copyright 2023, Elsevier Ltd. b) SEM images showing the microstructure of PLA/TPU/Fe_3_O_4_ (P/T/F) composites with varying Fe_3_O_4_ concentrations. Reproduced with permission.^[^ ^90^ ^]^ Copyright 2023, Elsevier Ltd. c) Shape memory performance of P/T/F composites, displaying the shape recovery ratio as a function of time. Reproduced with permission.^[^ ^90^ ^]^ Copyright 2023, Elsevier Ltd. d) Visualization of shape programming and sequential shape memory recovery of a bioinspired flower, captured through digital photographs and infrared thermal imaging.^[^ ^91^ ^]^ e) Conceptual diagram explaining the magneto‐responsive shape memory effect in the composites. Reproduced with permission.^[^ ^92^ ^]^ Copyright 2024, Elsevier Ltd. f) Geometric configuration of a reentrant structure, g) model slicing using CURA software, h) 3D‐printed components, i) compressed fixed shape of the reentrant structure with an inset force‐displacement curve, and j) magnetically triggered shape memory recovery process. Reproduced with permission.^[^ ^93^ ^]^ Copyright 2025, Elsevier Ltd. k) Shape memory response of composites containing varying Fe_3_O_4_ concentrations activated by a magnetic field. Reproduced with permission.^[^ ^94^ ^]^ Copyright 2025, Elsevier Ltd.

**Figure 9:**
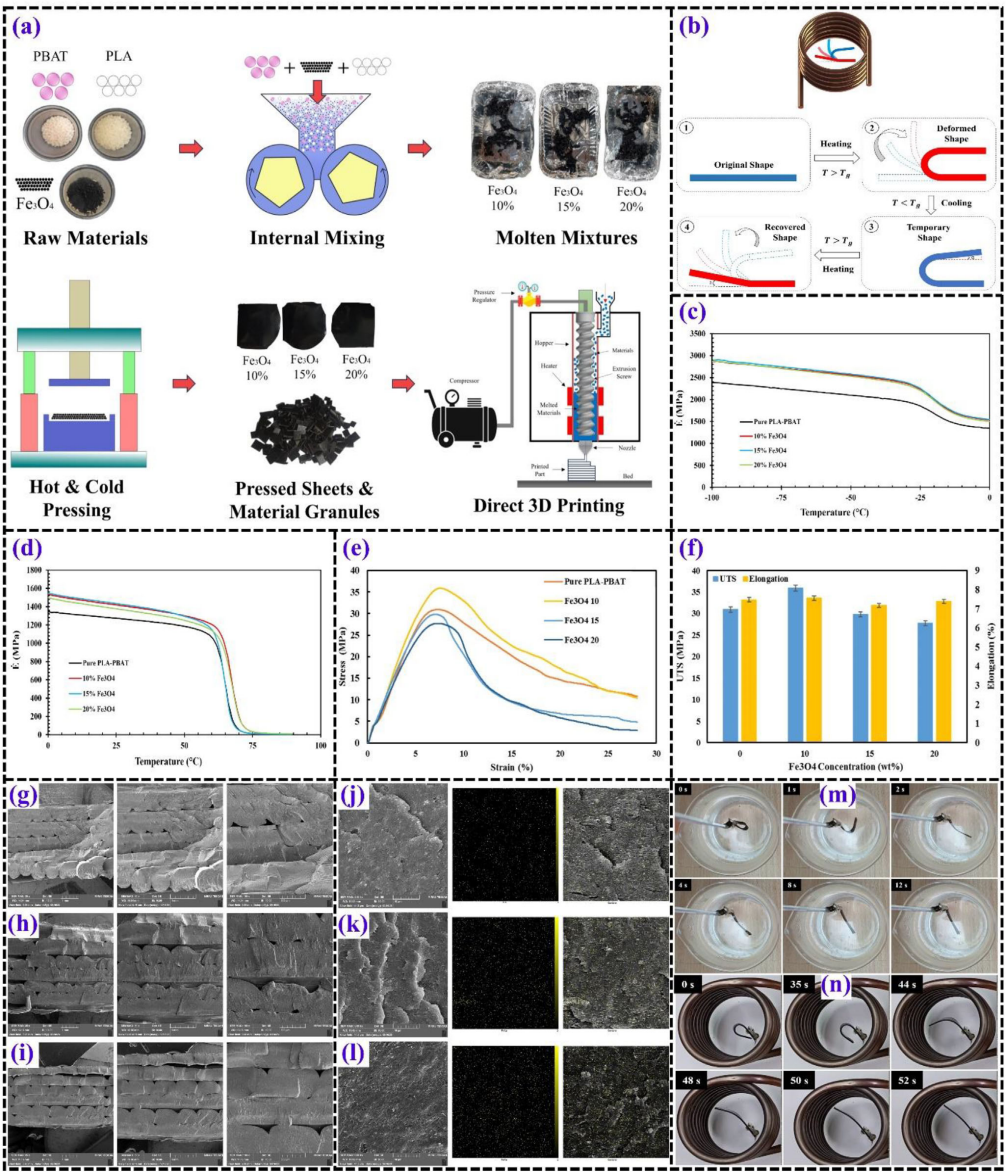
a) Sequential steps in the fabrication of 3D‐printed composite materials. b) Evaluation process for shape recovery behavior. Temperature‐dependent storage modulus variations at different Fe_3_O_4_ concentrations: c) ranging from −100 to 0 °C and d) from 0 to 100 °C. e) Stress‐strain curves illustrating mechanical responses for different Fe_3_O_4_ concentrations. f) Relationship between Fe_3_O_4_ content (wt%) and ultimate tensile strength (UTS) as well as uniform elongation. SEM imaging of PLA‐PBAT nanocomposites to assess printability at varying nanoparticle loadings: g) 10%, h) 15%, and i) 20%, captured at different magnifications (35×, 50×, and 100×). Morphological examination of PLA‐PBAT‐Fe_3_O_4_ nanocomposites with different magnetic nanoparticle contents: j) 10%, k) 15%, and l) 20%. m) Hot water bath‐induced shape recovery analysis for Fe_3_O_4_‐15%. n) Magnetic field‐driven shape recovery analysis for Fe_3_O_4_‐15%.^[^ ^96^ ^]^

Poly (lactic acid) or PLA is a biodegradable thermoplastic polymer derived from renewable resources such as corn starch or sugarcane. Unlike traditional petroleum‐based 3D printing filaments, PLA is sustainable and eco‐friendly. PLA offers excellent dimensional stability, minimizing warping during printing. However, it is brittle, making it less suitable for applications that require flexibility or impact resistance. Additionally, PLA degrades when exposed to UV light, limiting its durability outdoors. While not water‐soluble, it can dissolve in certain solvents like acetone and caustic soda. PLA is also classified as food‐safe, allowing its use in food containers and utensils, depending on its composition and processing standards. PLA's accessibility, environmental advantages, and user‐friendly nature make it a preferred choice for prototyping and low‐impact manufacturing.^[^ ^78^, ^79^ ^]^ Additionally, PLA exhibits a T_g_ ranging from 55 to 70 °C, which plays a crucial role in its shape memory behavior. Above this T_g_, PLA transitions from a rigid to a rubbery state, allowing for temporary shape fixation and recovery when cooled and reheated, making it a viable matrix for shape memory applications. The semi‐crystalline nature of PLA contributes to its shape memory properties, where crystalline domains act as anchor points stabilizing the original shape, while amorphous regions allow for deformation and recovery. This unique structure enables PLA‐based materials to undergo programmed shape transformations under external stimuli, particularly heat. To enhance its mechanical strength and responsiveness, PLA is often blended with soft polymers or reinforced with nanoparticles.^[^ ^80^ ^]^ **Figures** **5**, **6**, **7**, **8** and **9** illustrate the typical test results demonstrating the shape recovery behavior and magneto‐responsive performance of PLA‐based matrices.

##### PETG

**Figure 10:**
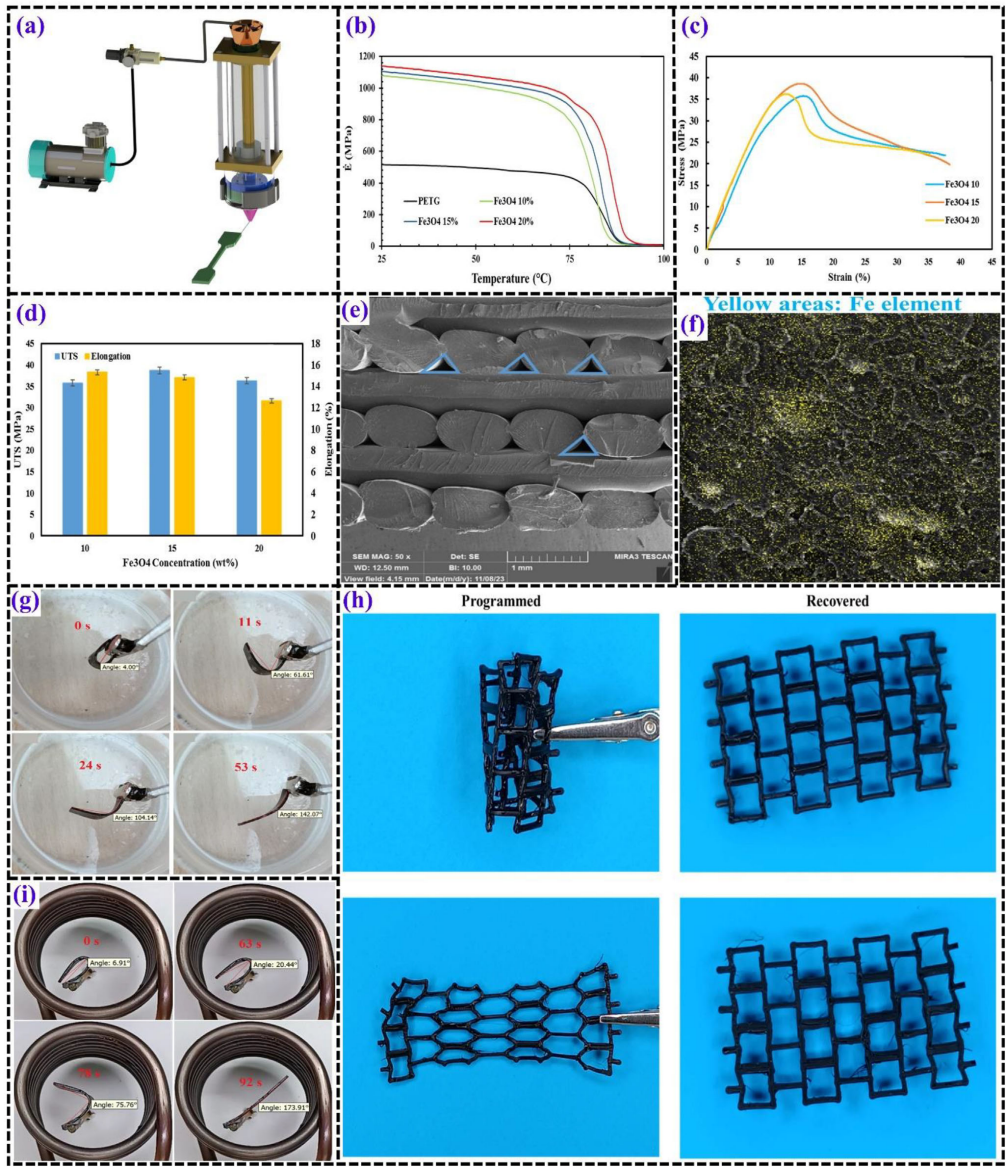
a) Schematic diagram illustrating the direct 3D printing process. b) DMTA results comparing pure PETG and PETG‐Fe_3_O_4_ nanocomposites. c) Engineering stress‐strain curves and d) extracted quantitative data from tensile testing of 3D‐printed PETG‐Fe_3_O_4_ nanocomposites. e) SEM micrographs of the fracture surface of a 3D‐printed PETG nanocomposite containing 15% Fe_3_O_4_. f) Elemental distribution (EDX mapping) of a PETG nanocomposite with 15% Fe_3_O_4_. g) Time‐dependent direct shape recovery process for a 3D‐printed PETG‐Fe_3_O_4_‐10 nanocomposite. h) PETG nanocomposite samples after shape programming and subsequent recovery. i) Time‐lapse visualization of the indirect shape recovery process in a 3D‐printed PETG‐Fe_3_O_4_‐10 nanocomposite.^[^ ^21^ ^]^

**Figure 11:**
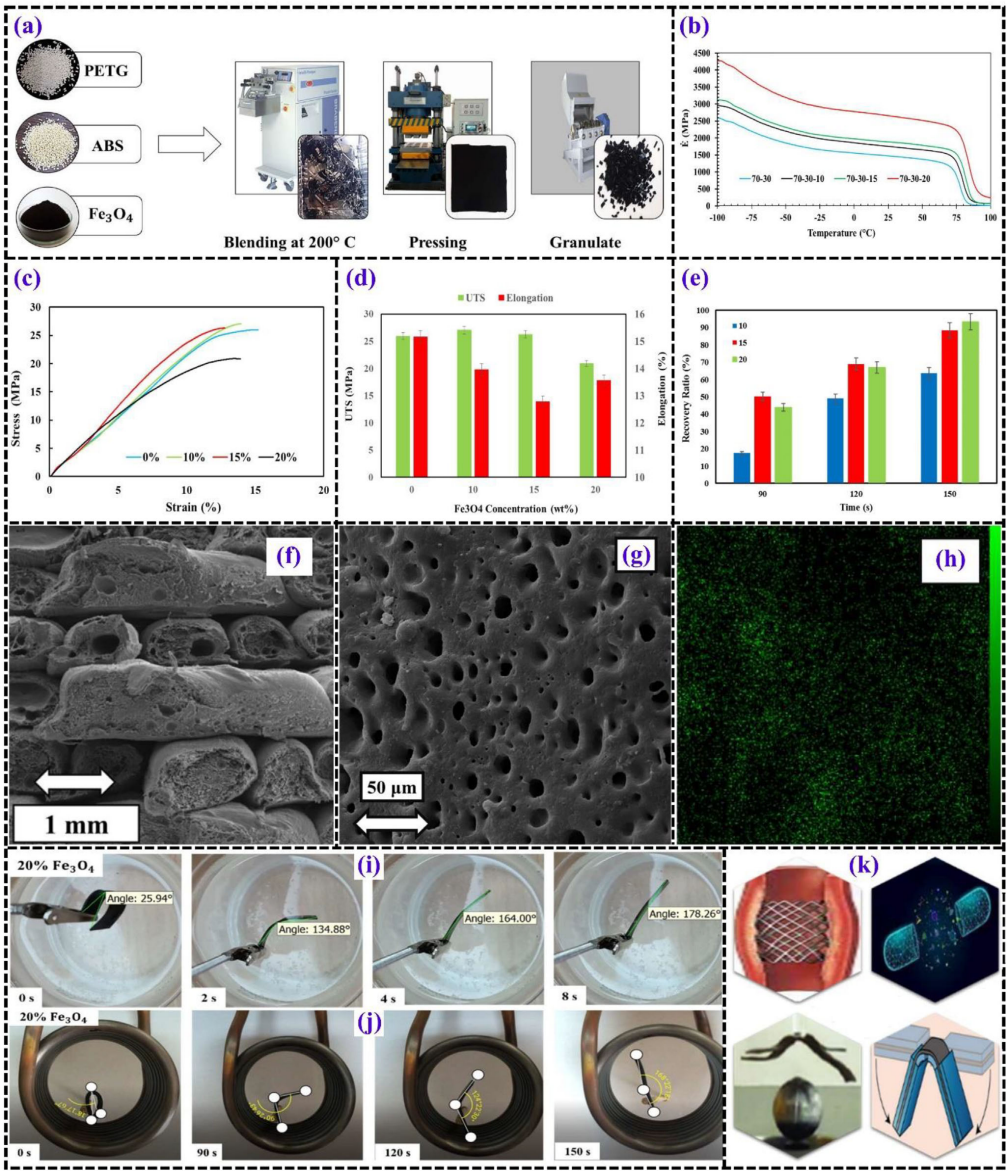
a) Sequential fabrication steps for PETG–ABS–Fe_3_O_4_ (P/A/F) nanocomposites. b) Thermal analysis results, including storage modulus variations in P/A/F composites. Tensile behavior of P/A/F nanocomposites at different Fe_3_O_4_ concentrations: c) stress‐strain curves and d) corresponding strength and elongation calculations. e) Time‐dependent shape recovery percentages of P/A/F nanocomposites under indirect stimulation. f) SEM images of fractured surfaces in printed specimens containing 20% Fe_3_O_4_. g) SEM micrograph illustrating the structural morphology and h) EDX mapping of P/A/F‐20%. i) Shape recovery process of P/A/F‐20% induced by: (i) direct thermal activation, and j) indirect stimulation. k) Demonstration of remote‐controlled applications of magnetic SMPs.^[^ ^99^ ^]^

Polyethylene terephthalate glycol (PETG) is a glycol‐modified version of the widely used thermoplastic, polyethylene terephthalate (PET), which is known for its enhanced properties suitable for a wide range of applications. The modification with glycol reduces the material's T_m_, making it easier to process and more user‐friendly compared to its unmodified counterpart. PETG is characterized by its excellent printability, which contributes to its growing popularity in 3D printing and industrial applications. Additionally, it exhibits impressive mechanical strength, allowing it to withstand significant stress while maintaining good chemical resistance and the ability to endure high temperatures. A notable feature of PETG is its minimal warping during the printing process, ensuring consistent and high‐quality results. PETG's T_g_ plays a critical role in its shape memory capabilities; the T_g_ for PETG is generally ≈80 °C, making it suitable for shape recovery processes at relatively low temperatures compared to other materials. While PETG offers various advantages, it does have some limitations. For instance, layer adhesion can sometimes be weak, leading to issues with the bond between printed layers. Stringing, the formation of thin filaments during the printhead's movement, can also pose challenges in certain prints. Beyond its thermal and mechanical properties, PETG also offers excellent UV resistance, making it a preferred material for outdoor applications and long‐term use, where durability against environmental factors is essential. However, PETG is immiscible with certain polymers, which can affect the blending of different materials for composite applications. In terms of post‐processing, PETG is soluble in solvents like toluene and methyl ethyl ketone (MEK), enabling intricate surface finishes and enhanced details in finished products. Furthermore, PETG's food‐safe properties extend its utility in the food and beverage industries, paralleling the characteristics of PET, while maintaining its strength and durability. These attributes collectively make PETG an attractive material choice, particularly in industries that require strong, durable, and versatile materials with the capability of 3D/4D printing applications.^[^ ^21^, ^87^, ^88^, ^89^ ^]^ **Figures** **10** and **11** present the test results from studies on magneto‐responsive PETG‐based SMPs.

##### PLA‐TPU

PLA and thermoplastic polyurethane (TPU) blends have garnered significant attention in 3D and 4D printing applications due to their complementary properties. PLA provides thermal stability, stiffness, and biodegradability, while TPU contributes high elasticity, toughness, and excellent shape memory performance. The T_g_ of these blends is critical for shape recovery behavior. For PLA‐TPU composites, two distinct T_g_ values are observed: ≈−20 °C for the TPU phase and 67 °C for the PLA phase. These dual T_g_ values enable tailored programming conditions, allowing for SME activation under various thermal stimuli. The mechanical properties of PLA‐TPU blends are influenced by the component ratio; for instance, blends with 70 wt.% PLA exhibits a maximum recovery stress of 12.85 MPa and a shape recovery ratio of 96.4% under compression loading. This optimal ratio balances the stiffness of PLA with the elasticity of TPU, ensuring efficient shape fixity and recovery. A notable characteristic of PLA‐TPU blends is their thermodynamic immiscibility, evidenced by dual storage modulus drops corresponding to each polymer's T_g_. SEM reveals TPU droplets dispersed within the PLA matrix, with larger TPU domains forming at higher TPU concentrations. This microstructural arrangement plays a pivotal role in determining the SME. The elasticity of TPU facilitates energy storage during deformation, enhancing shape recovery, while PLA's crystalline regions act as network points that anchor the temporary shape. Moreover, hot programming (in the rubbery region) yields the highest shape fixity ratios (up to 99%), whereas cold programming enhances recovery stress, showcasing the tunability of PLA‐TPU composites for various smart applications. The high processability of these blends further supports their use in FDM, enabling the creation of complex, customizable magneto‐responsive structures.^[^ ^90^, ^95^ ^]^

##### PLA‐PBAT

The PLA‐PBAT matrix presents a synergistic combination of mechanical resilience and flexibility, making it an ideal candidate for multifunctional applications. PLA, known for its biodegradability, high stiffness, and thermal stability, suffers from brittleness and low elongation at break. The incorporation of PBAT, a ductile aliphatic‐aromatic copolymer, significantly enhances the toughness and elasticity of the blend, overcoming the intrinsic rigidity of PLA. The T_g_ of the components plays a pivotal role in shape recovery performance. Specifically, PLA exhibits a T_g_ of ≈65–70 °C, while PBAT demonstrates a lower T_g_ near −15 °C, highlighting the immiscible nature of the blend due to a ≈90 °C T_g_ difference. This immiscibility results in a phase‐separated morphology, where PBAT domains are dispersed within the PLA matrix, forming a characteristic sea‐island structure. Such morphology, despite indicating limited interfacial adhesion, ensures enhanced shape memory behavior by providing multiple switching segments activated at distinct temperatures. This immiscible polymer blend exhibits a distinct phase‐separated morphology, with PBAT forming discrete domains within the PLA matrix, leading to an optimized balance between strength and ductility. Additionally, the presence of PBAT improves processability, reducing the need for external plasticizers while maintaining the material's environmental sustainability. The PLA‐PBAT matrix, therefore, offers a compelling combination of biodegradability, mechanical adaptability, and ease of fabrication, making it particularly suited for applications that demand high toughness and flexibility.^[^ ^96^, ^97^ ^]^

##### PETG‐ABS

The PETG‐ABS composite matrix combines the strengths of PETG and acrylonitrile butadiene styrene (ABS) to enhance 4D printing capabilities, especially using FDM. PETG offers excellent extrusion and shape memory capabilities, making it a popular choice for 3D and 4D printing. However, it has limitations, including a high relaxation rate after deformation due to molecular sliding, which can compromise its ability to retain strain and affect the stability of printed structures. The incorporation of ABS, which has a higher T_g_ of ≈110 °C compared to PETG's 80–85 °C, helps to address this limitation. ABS, with its more rigid structure, limits the mobility of PETG's molecular chains, reducing relaxation and enhancing the mechanical stability of the final product. As a result, this blend achieves a balance between the elasticity of ABS and the shape memory characteristics of PETG. The T_g_ difference between PETG and ABS also contributes to the formation of a phase‐separated morphology in the blend. This immiscibility results in distinct domains within the matrix, which are responsible for its enhanced mechanical properties. The mechanical strength of the PETG‐ABS composite is significantly improved over pure PETG, with the addition of ABS providing enhanced tensile strength and elongation properties. The PETG‐ABS composite also demonstrates superior shape memory behavior, especially in environments where both heat and mechanical stress are involved, ensuring efficient recovery to the original shape after deformation. The composite matrix, due to its improved printability and ability to adapt to multi‐stimulus environments, shows great promise for 4D printing, allowing for the creation of complex and responsive structures that can undergo controlled shape transformations. This combination of thermal stability, mechanical adaptability, and shape recovery properties makes PETG‐ABS a robust candidate for smart material applications in the 4D printing domain.^[^ ^98^, ^99^ ^]^

#### 3.3.2 Thermosets

##### AUD‐HEMA

**Figure 12:**
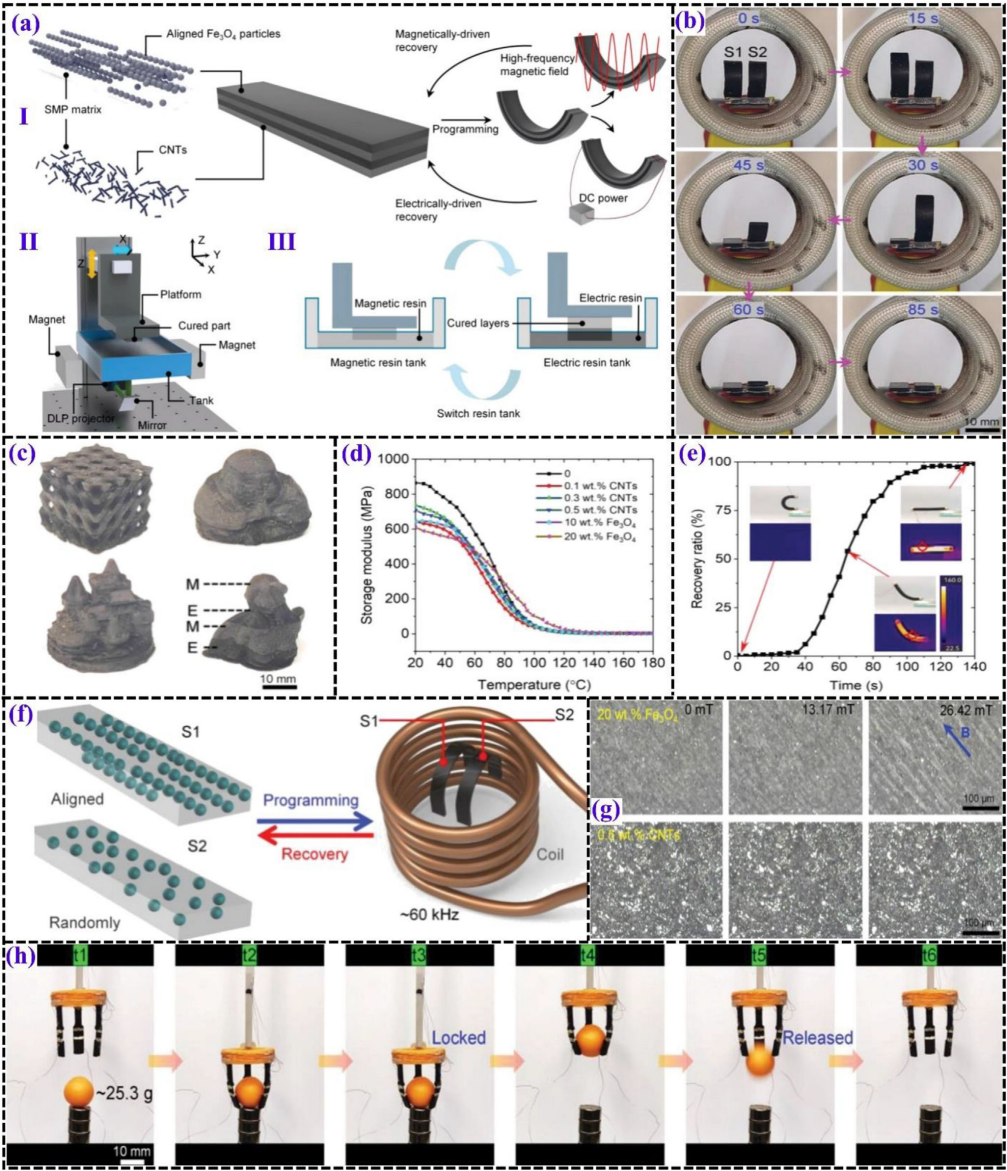
a) Principles of the design, shape memory mechanism, and fabrication process of ML‐EMSMCs using the multi‐material MF‐DLP 4D printing technique, including (I) a schematic illustration of the design concept and shape memory mechanism, (II) a diagram of the multi‐material MF‐DLP 3D printing system, and (III) the stepwise multi‐material 3D printing procedure. Reproduced with permission.^[^ ^100^ ^]^ Copyright 2024, Wiley‐VCH. b) Comparison of recovery speeds between particle‐aligned and randomly distributed samples, with the aligned configuration showing faster recovery performance. Reproduced with permission.^[^ ^101^ ^]^ Copyright 2025, Elsevier Ltd. c) Various complex 3D structures fabricated using the MF‐DLP 4D printing technique, demonstrating excellent printability and structural integrity. Reproduced with permission.^[^ ^100^ ^]^ Copyright 2024, Wiley‐VCH. d) Storage modulus behavior of the composites over a temperature range of 20–180 °C. Reproduced with permission.^[^ ^100^ ^]^ Copyright 2024, Wiley‐VCH. e) Electrically induced shape recovery and selective manipulation in a four‐layered structure, powered by a direct current of 200 V. Reproduced with permission.^[^ ^100^ ^]^ Copyright 2024, Wiley‐VCH. f) Magnetically driven shape recovery of ML‐EMSMCs with different electric/magnetic layer configurations, showing schematics of Fe_3_O_4_ particle alignment versus random distribution, and magnetic actuation within a high‐frequency alternating magnetic field. Reproduced with permission.^[^ ^101^ ^]^ Copyright 2025, Elsevier Ltd. g) Comparison of magnetic performance between 0.5 wt.% CNT‐loaded resin and 20 wt.% Fe_3_O_4_‐containing resin. Reproduced with permission.^[^ ^100^ ^]^ Copyright 2024, Wiley‐VCH. h) Sequential images capturing electrically and magnetically driven actuation processes in ML‐EMSMCs, highlighting rapid, programmable, and reversible shape transformations. Reproduced with permission.^[^ ^102^ ^]^ Copyright 2025, Wiley‐VCH.

**Figure 13:**
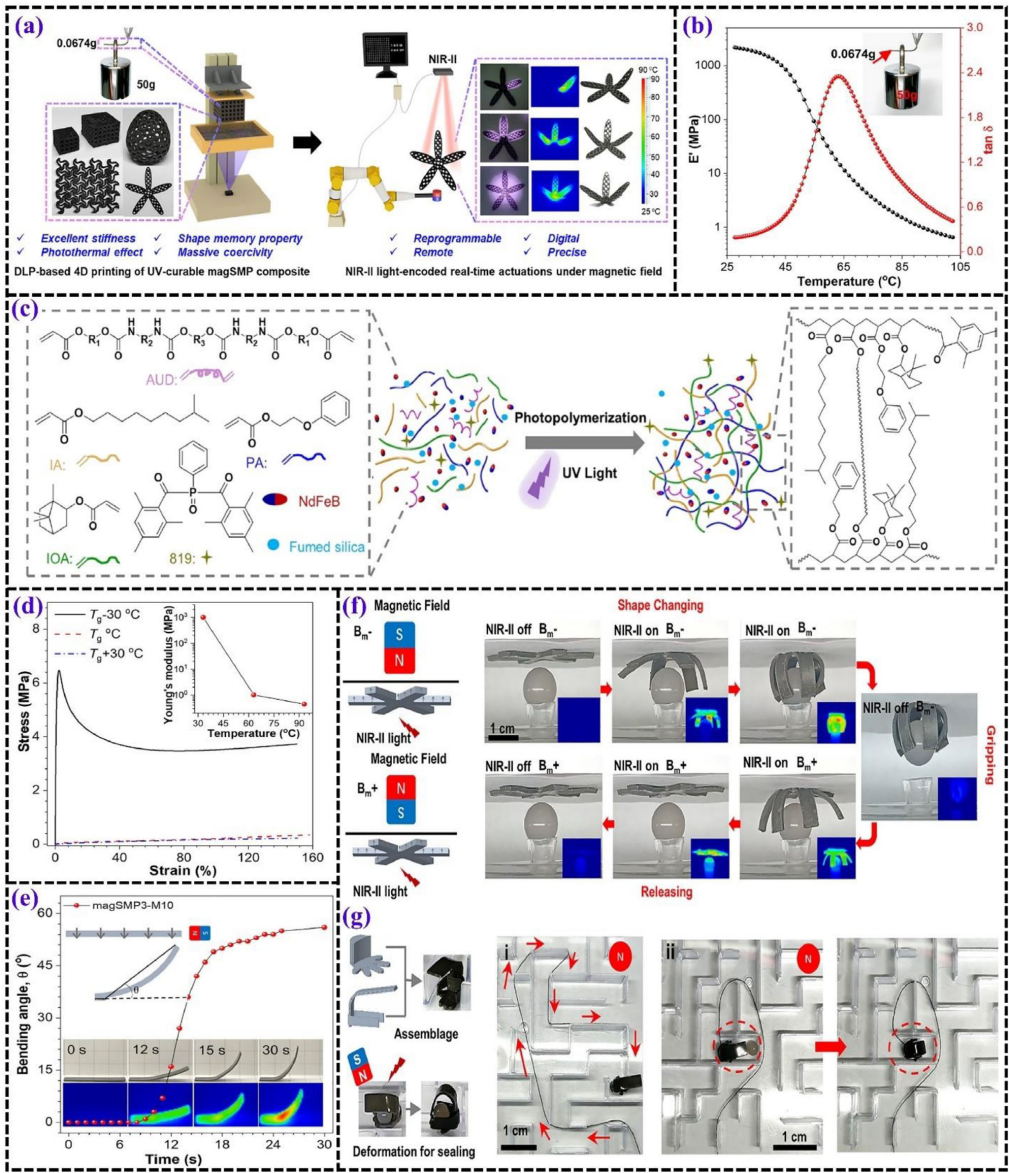
a) Schematic representation of a NIR‐II light‐encoded 4D‐printed magnetic shape memory composite (magSMP) designed for real‐time, reprogrammable soft actuators. b) Graph showing the relationship between storage modulus, tan δ, and temperature for the magSMP composite, with an inset displaying a locked bending cantilever supporting a weight. c) Illustration of the photopolymerization process and molecular structures of the magSMP precursor components. d) Tensile stress–strain curves of the magSMP film measured at temperatures of T_g_−30 °C, T_g_, and T_g_+30 °C. e) Plot of bending angle against irradiation time for the magSMP3‐M10 composite stripe, subjected to simultaneous NIR‐II light exposure (1064 nm, 0.6 W cm^−2^) and a magnetic field of 50 mT. f) Diagram depicting the magnetization of a six‐arm magSMP gripper and the orientation of the applied magnetic field, along with a demonstration of the gripper's ability to grip, lift, and release a ball when controlled by NIR‐II light (1064 nm, 0.3 mW cm^−2^) and a magnetic field of 110 mT. g) Sequential images showing a magSMP‐based robot navigating through a maze by following a flexible rope under magnetic guidance. Reproduced with permission.^[^ ^103^ ^]^ Copyright 2024, Elsevier Ltd.

A photosensitive SMP resin, primarily composed of aliphatic urethane diacrylate (AUD) as the cross‐linker, 2‐hydroxyethyl methacrylate (HEMA) as the monomer, and phenyl bis (2,4,6‐trimethylbenzoyl)‐phosphine oxide as the photoinitiator, serves as an effective base matrix for magnetic nanoparticle integration. The matrix exhibits a high T_g_ of ≈120 °C, a critical factor for reliable shape recovery performance under external stimuli. This elevated T_g_ ensures enhanced thermal stability, enabling the material to retain its structural integrity in high‐temperature environments. Mechanically, the matrix offers a storage modulus ranging from 863.5 to 600 MPa, depending on filler concentration. This temperature‐dependent variation in storage modulus reflects the polymer's molecular mobility, essential for reversible deformation and effective shape recovery. Notably, the matrix demonstrates compatibility with multi‐material magnetic field‐assisted digital light processing (MF‐DLP) 4D printing, allowing precise control over filler distribution and complex layer architectures. CNTs can be incorporated to enhance electrical conductivity, while Fe_3_O_4_ nanoparticles introduce magnetic responsiveness. Despite the inherent immiscibility between Fe_3_O_4_ nanoparticles and the polymer matrix, which may lead to the formation of micro‐interfaces and potentially reduce interlayer bonding strength, this challenge can be addressed by incorporating 3 wt.% silane coupling agent (KH 550). The addition of KH 550 enhances interfacial bonding strength by ≈60%, effectively preventing delamination and ensuring structural integrity. With its high thermal resistance, robust mechanical performance, and multi‐stimuli responsiveness, this matrix is well‐suited for the fabrication of advanced actuators, soft robotics, and multifunctional sensing devices in 3D and 4D printing applications.^[^ ^100^ ^]^ **Figures** **12** and **13** display the test results of UV‐curable SMP resin, thermosets fabricated using DLP.

##### IOA‐PA‐IA‐AUD

The base matrix employed for the incorporation of magnetic nanoparticles is a UV‐curable SMP resin, specifically formulated to support DLP‐based 4D printing. The matrix formulation includes isobornyl acrylate (IOA), 2‐phenoxyethanol acrylate (PA), and isodecyl acrylate (IA) as linear chain builders, with AUD acting as the cross‐linker. The T_g_ plays a pivotal role in the shape recovery performance of this matrix and can be precisely tailored by adjusting the IOA content. The T_g_ ranges from 37to 92 °C, with an optimized composition yielding a T_g_ of 65 °C, which ensures efficient reversible shape morphing and mechanical adaptability. The storage modulus reaches 1910 MPa at ≈35 °C, indicating robust mechanical integrity at lower temperatures. At elevated temperatures, the rubbery modulus decreases to 0.95 MPa, facilitating flexible deformation essential for shape‐memory applications. The mechanical properties of the matrix are distinguished by its exceptional tensile performance, accommodating elongations up to 160% across a temperature range of 35 to 95 °C. This degree of flexibility is critical for achieving large actuation movements required in soft robotic applications. The matrix also exhibits a R_f_ of ≈98% and a R_r_ of ≈92% over multiple cycles, underscoring its superior shape memory reliability. Compatibility with magnetic fillers, such as NdFeB particles, is enhanced by the inclusion of fumed silica, which prevents nanoparticle aggregation and ensures uniform dispersion throughout the polymer network. This homogeneity preserves the photothermal conversion efficiency (ηPT) at 42% under NIR‐II light irradiation and maintains high coercivity, essential for stable magnetic actuation. The phase homogeneity of the matrix ensures consistent mechanical and thermal performance, making it highly suitable for advanced multi‐modal 4D printing applications.^[^ ^103^ ^]^

### 3.4 Fillers

Among various magnetic iron‐based nanoparticles such as γ‐ Fe_2_O_3_,^[^ ^104^ ^]^ Fe_3_O_4_,^[^ ^105^ ^]^ CIP, NdFeB,^[^ ^106^ ^]^ and nickel zinc ferrite.^[^ ^23^ ^]^ Fe_3_O_4_ nanoparticles are particularly notable for their high magnetization, low cytotoxicity, and excellent biocompatibility. This section explores the potential of these nanoparticles in the 4D printing process for applications involving magnetic stimulation.

#### 3.4.1 Fe_3_O_4_

Iron oxide with the chemical composition Fe_3_O_4_ naturally exists as a magnetic mineral and appears as a black powder in laboratory settings. Nanoparticles of iron oxide, with their unique properties such as high surface‐to‐volume ratio, biocompatibility, and superparamagnetic characteristics, are widely used in applications such as water and wastewater treatment for removing pollutants, drug delivery, medical imaging, and designing catalysts for various organic reactions. The properties of Fe_3_O_4_ make it ideal for integration into magnetic‐actuated SMPs.^[^ ^107^, ^108^, ^109^ ^]^

#### 3.4.2 CIP

Carbonyl iron powder is a high‐purity form of iron produced by chemically decomposing purified iron pentacarbonyl. It typically appears as a grey powder consisting of spherical microparticles, with carbon, oxygen, and nitrogen as the primary impurities. Carbonyl iron powdered cores maintain stable parameters across various temperatures and magnetic flux levels, achieving excellent Q factors between 50 kHz and 200 MHz. They are commonly used in broadband inductors for high‐power applications due to their high permeability and low core losses, making them ideal for high‐frequency switching circuit output chokes and resonant inductors. Their fine particle size reduces eddy current losses, enhancing efficiency.^[^ ^110^, ^111^ ^]^

#### 3.4.3 NdFeB

NdFeB is a permanent magnet made from neodymium, iron, and boron, featuring a tetragonal Nd2Fe14B structure. They are widely used in cordless tools, hard drives, and magnetic fasteners, and can be sintered or bonded. Neodymium is antiferromagnetic below 19 K, but its iron compounds are ferromagnetic, with Curie temperatures above room temperature. Their tetragonal structure allows for magnetization along one axis, leading to greater strength. This has led to the replacement of alnico and ferrite magnets in high‐strength applications.^[^ ^112^, ^113^ ^]^

#### 3.4.4 Nickel‐Zinc Ferrite

This material is produced through a reverse‐micellar microemulsion process, showcasing distinctive electric, magnetic, and thermal properties. This makes it suitable for various applications such as catalysts, absorbents, gas sensors, and cancer treatment. With permeabilities below 2500 and high resistivity (super passing MnZn), it is ideal for use in high‐temperature applications ranging from 1 to 2 MHz and several hundred MHz. Structurally, Ni‐Zn ferrites are quaternary compounds with a face‐centered cubic lattice of oxygen anions, following the formula X‐Fe_2_O_3_, where X is a divalent metal cation like Ni, Zn, Co, or Mn.^[^ ^114^, ^115^ ^]^

## 4 Review

The choice of matrix polymer plays a decisive role in defining the shape memory behavior, mechanical robustness, processing feasibility, and suitability of magneto‐responsive SMPs for target applications such as soft robotics, biomedical devices, and wearable technologies. This section discusses key thermoplastic and thermoset matrices used in magnetic SMP systems, drawing comparisons between their shape memory performance, actuation characteristics, and 4D printing compatibility.

### 4.1 Thermoplastics: Processable, Biocompatible, and Tunable

Thermoplastics are widely favored for 4D printing due to their reprocessability, cost‐effectiveness, and compatibility with extrusion‐based methods. Among them, PLA, PETG, and PCL, as well as their blends, stand out for their diverse mechanical and shape memory properties.

1. PLA is recognized for its excellent printability and biodegradability, offering a low glass transition temperature (T_g_ ≈60 °C), which facilitates low‐temperature actuation. Its semi‐crystalline structure allows good shape fixity and recovery, although its brittleness and limited strain tolerance restrict its use in load‐bearing or dynamic applications. Nevertheless, it is ideal for temporary implants, smart fluidic systems, and bioinspired structures (actuators) where moderate SME performance, low‐force actuation, biodegradability, and biocompatibility are prioritized.
2. PETG provides superior ductility, printability, and minimal warping, making it particularly suitable for high‐speed fabrication of complex geometries with magnetic fillers. Unlike PLA, PETG exhibits enhanced strain tolerance at ambient and elevated temperatures and demonstrates excellent shape recovery (Rr ≈100%) even under cold programming. Its higher T_g_ (≈80 °C) and robustness make it well‐suited for adaptive clothing, deployable robotics, and remote‐controlled wearable medical devices, where durability and repeatability are essential.
3. PCL, with a much lower T_g_ (≈−60 °C) and melting point (≈60 °C), offers high flexibility and biodegradability. While its shape memory performance is relatively weaker, due to crystalline switching governed by T_m_, its exceptional biocompatibility and controlled degradation make it a preferred matrix for bone tissue engineering, drug delivery systems, and minimally invasive surgical tools and stents.
4. PLA‐based blends such as PLA‐TPU, PLA‐PBAT, PLA‐PMMA and etc. combine the rigidity and shape memory strength of PLA with the elasticity and toughness of TPU or PBAT. For instance, PLA‐TPU blends achieve high shape fixity (R_f_ ≈99%) and recovery (R_r_ >96%) with tunable thermomechanical behavior depending on the mixing ratio, enabling precise control over the actuation behavior. These blends are especially promising for wearable biosensors, compression garments, and self‐healing robotic elements, where elasticity, recovery force, and repeated cycling are required. PLA‐PBAT, with its enhanced toughness and flexibility, is better suited for deployable structures and dynamic soft robotic limbs, particularly in environmentally sensitive or sustainable applications, as it is a biodegradable composite.
5. PETG‐ABS composites strike a balance between PETG's printability and ABS's mechanical strength and thermal resistance. This blend minimizes relaxation post‐deformation and improves stability, making it ideal for high‐performance industrial actuators, protective smart textiles, and reusable robotic joints.
6. Finally, PHB‐PCL blends leverage PHB's thermal stability and PCL's softness to create dual‐segment SMPs responsive to distinct temperatures. Their biodegradable nature, tunable stiffness, and compatibility with magnetic fillers position them well for eco‐friendly biomedical scaffolds, minimally invasive stents, and precision surgical tools.

### 4.2 Thermosets: Structurally Robust and Photocurable

Thermoset SMP matrices, typically employed via vat photopolymerization, are highly crosslinked systems offering excellent mechanical integrity and shape stability. Although they lack the reprocessability of thermoplastics, their capacity for intricate, high‐resolution printing and tailored multi‐stimulus responsiveness makes them indispensable for precision applications.

AUD‐HEMA and IOA‐PA‐IA‐AUD‐based systems are representative UV‐curable matrices that exhibit high T_g_ (up to 120 °C), robust tensile strength, and good elasticity. These characteristics support their use in soft robotics, multi‐stimulus actuators, minimally invasive surgical tools, and magnetically actuated microgrippers. Their compatibility with multi‐material DLP printing allows layer‐specific mechanical tuning and integration of sensing/actuation components.

Despite their typically brittle nature, thermosets used in magnetically responsive systems can be optimized with toughening agents and elastomeric monomers, achieving a compromise between stiffness and actuation range. Their superior photothermal responsiveness and structural fidelity make them ideal for bio‐inspired actuators, soft sensors, and high‐resolution smart wearables where feature complexity and magnetic response precision are critical.

In summary, thermoplastics offer scalability, versatility, and moderate SME performance with the added benefits of recyclability and biocompatibility, making them ideal for mass‐customized biomedical devices and dynamic wearables. Thermosets, in contrast, support high‐resolution structures and multi‐material fabrication crucial for smart robotics and micro‐scale medical tools. The selection of matrix resin must therefore be guided not only by printability or recovery ratio but by the interplay between stimulus conditions, mechanical demands, and long‐term application stability. Furthermore, several thermoplastics such as PLA, PHB, and PCL offer biodegradability and eco‐friendly disposal, while materials like PETG support waste reduction through reliable printability and reusability. These characteristics, especially when applied to biomedical and wearable technologies, contribute to both environmental sustainability and resource‐efficient fabrication. **Table** **2** provides a detailed summary of research on different composites used in 4D printing applications that rely on magnetic actuation. It highlights the diverse matrix materials and fillers used in these composites, providing insights into how their properties have been modified or enhanced to achieve desired functionalities. This compilation serves as a resource for understanding the relationship between material composition and performance under magnetic stimuli.

**Table 2:** An overview of magneto‐responsive composites which developed in the literature.

Matrix	Fillers	Content (Volume Fraction)	Printing Mechanism	Highlights	Refs.
PLA	Fe_3_O_4_	90–10 85‐15 80‐20	FDM	Achieve a R_r_ of 95.6% and an R_f_ of 96.7% for 15%‐Fe_3_O_4_ composite Recovery time of 10 s by a magnetic field at 27.5 kHz	[83]
PLA	Fe_3_O_4_	85–15	FDM	Optimize bioinspired tracheal scaffolds for trachea geometry fitting Achieve shape recovery in 30 s under a 30 kHz magnetic field	[86]
PLA	Fe_3_O_4_	92–8 88‐12 82‐18	FDM	Develop tracheal stents using a curved rectangle with an S‐shaped hinge structure Achieve shape recovery within 40 s under a magnetic field with an R_r_ over 99%	[87]
PLA	Fe_3_O_4_	95‐5 90‐10 85‐15 80‐20	FDM	10% Fe_3_O_4_ composite exhibited a rapid R_r_ within 22 s under a magnetic field, with a R_r_ of ∼95%, and maintained >90% Rf after 5 cycles. Tensile strength of 10 wt.% Fe_3_O_4_ composite reached ≈58 MPa and remained stable through 8 weeks of degradation.	[116]
PLA	Fe_3_O_4_	85–15	FDM	Samples were magnetized during printing at 0°, 45°, and 90° relative to the magnetic field using various magnet placements. Tensile strengths of 13.96 MPa at 0° angle, indicating stronger printed lines over adhesion areas	[117]
PLA	metal powder	89‐21	FDM	A small magnetic field enables actuator modulation, achieving a maximum bending angle of 59° under low‐field conditions. Rapid shape recovery is demonstrated under controlled temperature and magnetic fields, powered by a 120 V supply.	[118]
PETG	Fe_3_O_4_	90–10 85‐15 80‐20	FDM	Achieving 96%–97% R_r_ under magnetic stimulation Recording maximum tensile strength of 38.66 MPa at 15% Fe_3_O_4_	[21]
PCL	Fe_3_O_4_	90–10 85‐15	Material extrusion	Tracheal scaffolds fabricated by material extrusion 3D printing using dynamically crosslinked nanoparticles. Achieving Tensile Strength of 59.70 MPa and Elastic Modulus of 24.68 MPa at 15 wt.% Fe_3_O_4_	[119]
PLA‐TPU	Fe_3_O_4_	0, 15, 20, 25, and 30 wt.% Fe_3_O_4_	FDM	Design and print innovative honeycomb and bionic flower‐like structures Demonstrate ∼100% R_f_ and >91% R_r_	[90]
PLA‐TPU	Fe_3_O_4_‐ Continuous carbon fiber (CCF)	85–15 (25 wt.% Fe_3_O_4_)	FDM	Designed and 4D‐printed re‐entrant and gripper structures exhibiting repeatable cold‐programmed shape recovery. Achieved >90% R_r_ under magnetic stimulation. Realized 22 s rapid recovery under combined electric and magnetic fields.	[120]
PLA‐PBAT	Fe_3_O_4_	PLA‐PBAT (70‐30)‐with 10%, 15%, and 20% Fe_3_O_4_	FGF	All composites achieved nearly 100% R_r_ under a magnetic field. Recovery began at 44 s (full at ≈64 s) for 10% Fe_3_O_4_; 15% and 20% started at ≈40 s (full at ≈54–56 s). All samples maintained high Rf. 10% Fe_3_O_4_ had the best mechanical properties: 16% higher UTS (35.89 MPa) and 15% greater toughness; higher loadings reduced strength due to agglomeration.	[96]
PLA‐PMMA	Fe_3_O_4_	PLA‐PMMA (50‐50)‐ Fe_3_O_4_(10%, 12%, and 15%)	FGF	Achieve 100% R_r_ in ≈85 s, with faster response noted at higher Fe_3_O_4_ content, particularly 15 wt.%.	[121]
PLA‐PPCU	Fe_3_O_4_	‐	FDM	Increasing elongation at break from 4% to 274% by incorporating renewable poly(propylene carbonate) polyurethane (PPCU)	[122]
PETG‐ABS	Fe_3_O_4_	PETG‐ABS(70‐30)‐ with 10%, 15%, and 20% Fe_3_O_4_	FGF	Achieve an increasing UTS to 27.05 MPa with elongation decreasing to 12.78% at 15 wt.% Fe_3_O_4_ Exhibit a superior shape‐memory performance under direct heat and magnetic stimulation at 20 wt.% Fe_3_O_4_	[99]
PHB‐PCL	Fe_3_O_4_‐cellulose nanofibers (CNFs)	PHB/PCL (80:20) with 10 wt.% Fe_3_O_4_ and 0.5 wt.% CNFs	FDM	Fabricate a scaffold with load‐carrying performance Achieve optimal strengthening and toughening effects at 10 wt.% Fe_3_O_4_ and 0.5 wt.% CNFs	[123]
TPU‐PCL	CIP	70‐30 80‐20 90‐10	FDM	Design thermally self‐recoverable periodic cellular structures with a CIP content of 30 wt.%. Record stiffness values of 57 kN m^−1^ at 25 °C and 3.92 kN m^−1^ at 65 °C in negative Poisson's ratio structure Analyze magneto‐deformation across temperatures: minimal bending below 20° at 25 °C, enhanced flexibility at 65 °C	[124]
PLMC‐PTMC	Fe_3_O_4_	PLMC‐PTMC (50‐50)‐Fe_3_O_4_ (20 wt.%)	DIW	Fabricate complex multi‐material structures with locally controllable deformations through dual‐stimuli interactions between thermal and magnetic triggers	[125]
PMMA‐TPU	Fe_3_O_4_	PPMA‐TPU (70‐30)‐ Fe_3_O_4_ (10%, 15%, and 20%)	FGF	Achieving 100% R_r_ with a UTS of 53.8 MPa at 10 wt.% Fe_3_O_4_ Enhancing tensile strength by 10%–15% with Fe_3_O_4_ nanoparticles while decreasing strain at break from 17% to 14%	[126]
AUD‐HEMA	Fe_3_O_4_ and CNTs	20% Fe_3_O_4_ and 0.5% CNTs	DLP	R_f_ ≈96%, R_r_ ≈97%. T_g_ enhanced to 120 °C. Storage modulus up to 863.5 MPa. Conductivity up to 5.37×10^−3^ S cm^−1^. Magnetization strength of 10.7 emu g^−1^. 8‐layer sample recovers 1.66× faster than 2‐layer sample under magnetic actuation. Dual actuation (electric Joule heating and high‐frequency magnetic field).	[100]
IOA‐PA‐ IA‐ AUD	NdFeB	0–15 vol% (selected optimal: 10 vol%)	DLP	R_f_ ≈ 98%, R_r_ ≈ 92%. Tunable T_g_ from 37 to 92 °C; optimized at T_g_ ≈ 65 °C. Storage modulus ≈1910 MPa at 35 °C; high tensile elongation. NdFeB particles achieved ≈42% photothermal conversion efficiency under NIR‐II (1064 nm) light. High coercivity enabled actuation under magnetic fields up to 50 mT. Rapid, reversible shape morphing under combined NIR‐II and magnetic stimulation.	[103]
Photocurable polymeric resins (acrylate‐based amorphous polymers)	NdFeB and Fumed silica nanoparticles	15 vol% NdFeB, 12–14 wt.% silica nanoparticles	DIW	Tunable Poisson's ratio and shear deformation + Shiftable mechanical behaviors (expansion, contraction, shear, bending) T_g_ ≈66 °C for M‐SMP Young's modulus: 1.16 GPa (22 °C) to 2.02 MPa (90 °C) for M‐SMP	[127]
Polycaprolactone dimethacrylate (PCLDMA)	Fe_3_O_4_	1.0, 2.5, and 5.0 wt.%	DLP	100% recovery at 5% filler within 2 min under a magnetic field (4 kA m^−1^, 375 kHz). Elastic modulus increased from 108 ± 5 MPa (pure PCLDMA) to 278 ± 26 MPa (5 wt.% composite) below Tm. Slight decrease in T_m_ with increased filler content (up to 74 °C at 5 wt.%). Uniform distribution of nanoparticles with minimal agglomeration (200–300 nm clusters).	[49]

## 5 Applications and Future Trends

Magnetically responsive SMPs, utilizing the capabilities of 4D printing, are rapidly emerging as a transformative material system with significant potential across diverse fields, particularly in biomedicine. Their unique ability to combine remote magnetic actuation with advanced design flexibility positions them as ideal candidates for a variety of innovative applications. In bone fracture repair, porous SMP scaffolds embedded with superparamagnetic nanoparticles, such as Fe_3_O_4_, are revolutionizing bone regeneration. They offer customizable mechanical strength, porosity, and biodegradability, promoting controlled healing and minimizing rejection risks. The integration of Fe_3_O_4_ allows for non‐invasive, magnetically triggered shape recovery, which accelerates healing trajectories and facilitates implant functionality. Beyond skeletal repair, the ability of Fe_3_O_4_ nanoparticles to induce hyperthermia under alternating magnetic fields holds promise for targeted cancer therapies. Additionally, magnetically actuated SMPs show potential in precise, localized drug delivery, enhancing treatment efficacy and reducing systemic impacts. Furthermore, remote activation of shape recovery expands telemedicine possibilities by enabling safer and more efficient remote medical procedures, as well as the development of wearable devices. In fabrication, refined FDM techniques involve using different concentrations of magnetic nanoparticles to achieve impressive SME. However, challenges arise in print quality and material properties when higher nanoparticle loadings are used.

**Figure 14:**
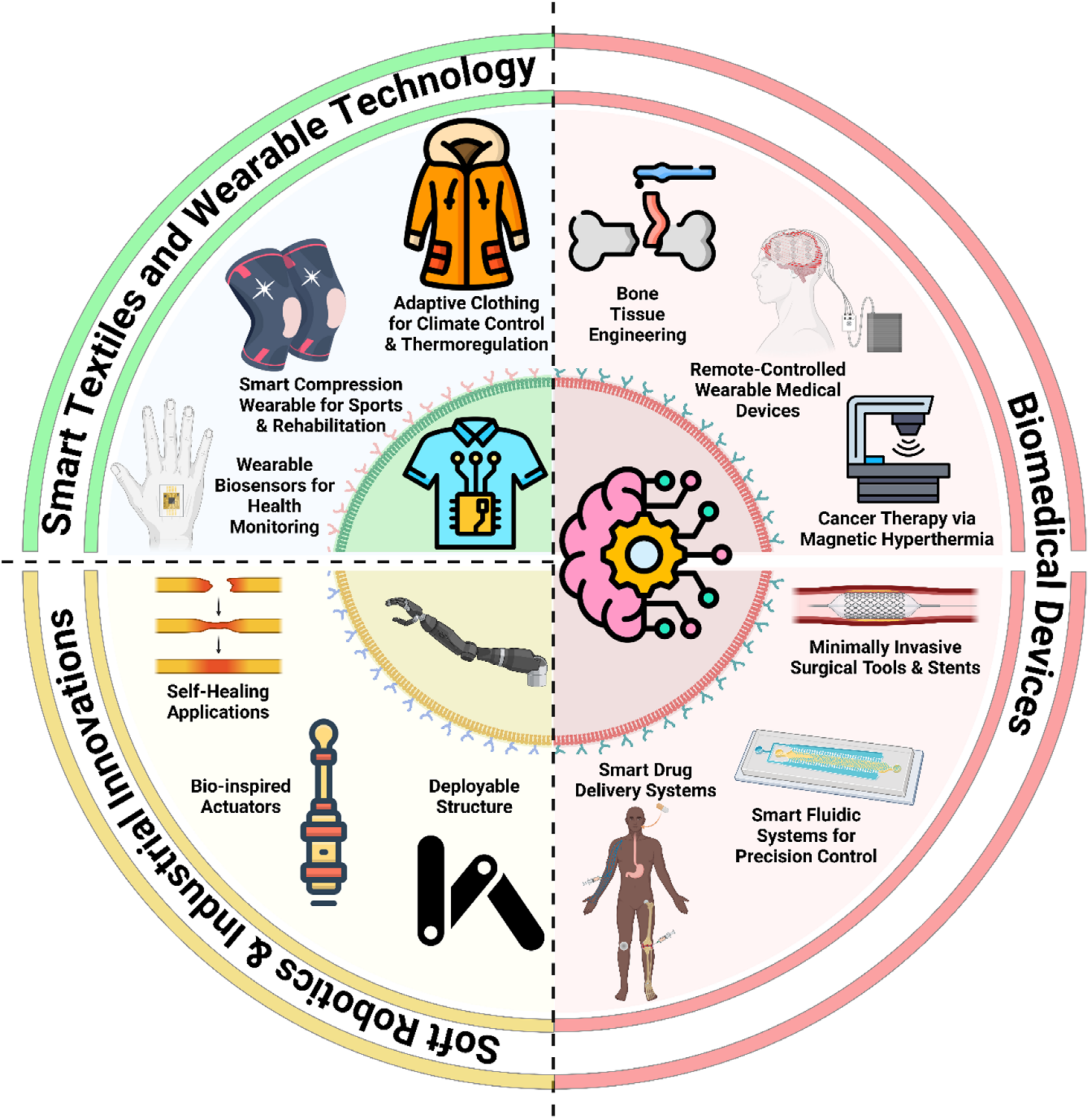
Schematic illustration of potential applications for magneto‐responsive SMPs.

Future trends include ongoing research focused on refining SMP matrices and integrating nanoparticles to improve material properties, print quality, and biocompatibility. The development of sophisticated control methods for magnetic actuation, which allow for precise tuning of shape recovery rates and forces, will enable optimized remote actuation with improved temporal and spatial control. This includes the integration of multi‐functional nanoparticles that combine diagnostic and therapeutic capabilities. The scope of magnetic SMPs is expanding into aerospace, allowing for adaptive structures to be used in space exploration. By utilizing the seamless synergy between functionality and biocompatibility, magnetically responsive SMPs are positioned to become pivotal materials for the next generation of advanced biomedical devices, systems, and other cutting‐edge technologies. **Figure** **14** illustrates an overview of magneto‐responsive SMPs’ potential applications.

### 5.1 Biomedical Applications

#### 5.1.1 Bone Tissue Engineering

One of the most promising applications of magneto‐responsive SMPs lies in bone tissue engineering and fracture repair. Conventional implants often require invasive surgeries, long recovery periods, and potential complications due to rigid structures that may not conform to dynamic biological environments. 4D‐printed biodegradable scaffolds with Fe_3_O_4_ nanoparticles offer an advanced alternative by allowing for non‐invasive shape recovery upon magnetic stimulation. These scaffolds can be compressed and inserted through small incisions, reducing trauma during implantation. Upon exposure to an alternating magnetic field, the Fe_3_O_4_ nanoparticles generate localized heat, softening the SMP matrix and triggering shape recovery to conform to the defect site. The structure then hardens as it cools, providing mechanical support while gradually degrading and being replaced by natural bone tissue.^[^ ^83^, ^128^, ^129^ ^]^ This technology eliminates the need for secondary removal surgeries, accelerates healing, and allows for patient‐specific customization, significantly improving orthopedic treatments.

#### 5.1.2 Cancer Therapy via Magnetic Hyperthermia

Beyond structural regeneration, magneto‐responsive SMPs are revolutionizing cancer therapy through magnetic hyperthermia. Traditional cancer treatments such as chemotherapy and radiotherapy often cause significant damage to surrounding healthy tissues. The integration of Fe_3_O_4_ nanoparticles into SMP implants enables a localized heating effect, wherein exposure to an alternating magnetic field generates controlled temperatures (43–45 °C), selectively inducing apoptosis in tumor cells while sparing healthy tissue. Unlike conventional heating methods, this precise thermal actuation provides a minimally invasive approach for tumor ablation, eliminating the need for repeated surgical interventions. Furthermore, these SMP implants can be designed to release embedded drugs simultaneously, combining hyperthermia with chemotherapy to enhance therapeutic efficacy.^[^ ^130^, ^131^, ^132^, ^133^ ^]^ This approach enhances treatment selectivity, reduces systemic toxicity, and improves overall patient outcomes.

#### 5.1.3 Smart Drug Delivery Systems

Another significant biomedical advancement is magnetically actuated drug delivery systems, which enable on‐demand, site‐specific release of therapeutic agents inside the body. Traditional drug administration methods often suffer from low bioavailability, systemic side effects, and poor patient compliance. By embedding Fe_3_O_4_ nanoparticles within a 4D‐printed SMP matrix, external magnetic stimulation can induce localized structural changes, opening controlled microchannels or pores to release medication at specific target sites.^[^ ^134^, ^135^, ^136^ ^]^ This mechanism ensures precise dosing, prevents overdosing risks, and enhances the effectiveness of time‐sensitive treatments. Unlike conventional polymer‐based controlled‐release systems, which rely on slow diffusion or enzymatic degradation, magnetically responsive SMPs offer tunable release rates, allowing dynamic control over treatment schedules and reducing the need for frequent drug administration.

#### 5.1.4 Remote‐Controlled Wearable Medical Devices

In the field of wearable medical devices and rehabilitation, magneto‐responsive SMPs are being utilized to develop remotely adjustable exoskeletons, compression garments, and assistive orthopedic braces. These materials enable smart, self‐adapting supports that conform dynamically to a patient's movements, providing real‐time adjustments in fit and support levels.^[^ ^137^, ^138^ ^]^ For example, an SMP‐based rehabilitation brace can be designed to remain flexible during movement and stiffen upon magnetic activation, offering variable support during different recovery phases. This adaptive functionality enhances patient comfort, compliance, and overall rehabilitation outcomes, reducing the dependency on multiple devices and manual adjustments.

#### 5.1.5 Minimally Invasive Surgical Tools and Stents

Magneto‐responsive SMPs are emerging as a transformative solution in minimally invasive surgical tools and deployable stents, offering a non‐invasive, remotely controlled alternative to traditional rigid implants. Conventional stents and surgical devices often require manual expansion or catheter‐based inflation, which poses risks of misalignment, tissue damage, and patient discomfort. In contrast, 4D‐printed SMP stents embedded with Fe_3_O_4_ nanoparticles can be inserted in a compact form and expanded remotely via an external magnetic field. Upon exposure, the localized heat generation softens the material, allowing the stent to expand and conform precisely to the arterial walls, ensuring secure placement and enhanced blood flow restoration. The programmable shape memory of these devices allows for customized mechanical properties, ensuring that expansion forces are sufficient to keep vessels open without excessive strain on surrounding tissues. Additionally, biodegradable variants of these stents remove the need for follow‐up surgeries, improving patient recovery and reducing long‐term complications. Beyond vascular applications, magneto‐responsive SMPs can be used in remotely actuated surgical retractors and grippers, allowing surgeons to manipulate tissues with higher precision without direct manual force, thereby reducing surgical trauma and post‐operative recovery times.^[^ ^87^, ^139^, ^140^ ^]^ These advancements are particularly beneficial in cardiovascular interventions, endoscopic procedures, and neurosurgery, where delicate and precise control is critical.

#### 5.1.6 Smart Fluidic Systems for Precision Control

The incorporation of magneto‐responsive SMPs in smart valves and microfluidic devices enables precise, programmable liquid and gas flow control in biomedical, chemical, and industrial processing systems. Unlike traditional mechanical valves that require external actuators or pressure differentials, 4D‐printed magneto‐responsive valves can be remotely adjusted via external magnetic fields, allowing for dynamic reconfiguration based on process requirements. These innovations are particularly beneficial in lab‐on‐a‐chip devices, biomedical diagnostics, and controlled drug delivery systems, where precise, real‐time modulation of fluid pathways is critical. By reducing mechanical complexity, improving response time, and enhancing operational efficiency, magneto‐responsive fluidic systems represent a next‐generation solution for automation and precision engineering.^[^ ^141^, ^142^, ^143^ ^]^

### 5.2 Soft Robotics and Industrial Innovations

#### 5.2.1 Deployable Structures

Magneto‐responsive SMPs are also poised to revolutionize engineering, particularly in deployable structures for applications. Traditional satellite components and expandable space modules rely on mechanical actuators, which add weight, complexity, and failure risks. By utilizing 4D‐printed magneto‐responsive materials, these structures can be compactly stored during launch and then remotely deployed in space using an external magnetic field. The controlled expansion mechanism triggered by Fe_3_O_4_ nanoparticles ensure accurate, repeatable shape recovery, making them ideal for self‐deploying antennas, morphing wings, and lightweight space habitats.^[^ ^144^, ^145^, ^146^ ^]^ This technology not only reduces payload weight and energy consumption but also extends mission longevity by eliminating mechanical wear and failure risks.

#### 5.2.2 Bio‐Inspired Actuators

Another critical application is in soft robotics and bio‐inspired actuators, where magnetically responsive 4D‐printed materials provide unprecedented flexibility and adaptability. Unlike traditional rigid robotic mechanisms, soft robots benefit from programmable deformation and shape recovery, enabling natural, human‐like movement for applications in surgical robotics, search‐and‐rescue operations, and industrial automation.^[^ ^147^, ^148^, ^149^, ^150^ ^]^ Magnetically actuated SMPs allow for remote, contactless control, enabling robots to reconfigure their structure in response to environmental demands. This advancement enhances precision, efficiency, and safety, particularly in minimally invasive surgery and micro‐manipulation.

#### 5.2.3 Self‐Healing Materials for Enhanced Durability

Beyond adaptive sealing, magnetically activated self‐healing materials provide a groundbreaking approach to damage repair in high‐performance applications. These materials utilize magnetic heating to trigger localized softening, allowing micro‐cracks and deformations to autonomously close and re‐establish structural integrity. This functionality is especially valuable in extreme environments, such as high‐pressure engines and corrosive chemical settings, where seal failures or structural defects can lead to catastrophic breakdowns.^[^ ^151^, ^152^, ^153^, ^154^, ^155^ ^]^ By embedding Fe_3_O_4_ nanoparticles, these materials can be stimulated externally, eliminating the need for manual repair and significantly improving system reliability and longevity.

### 5.3 Smart Textiles and Wearable Technology

#### 5.3.1 Adaptive Clothing for Climate Control and Thermoregulation

Magneto‐responsive SMPs are transforming wearable technology by enabling clothing that dynamically adjusts insulation in response to environmental changes or body temperature fluctuations. Unlike traditional garments that require manual layering or battery‐powered heating, these 4D‐printed fibers embedded with Fe_3_O_4_ nanoparticles can expand or contract upon magnetic stimulation, modifying air‐trapping layers to provide real‐time climate control. In cold environments, the material contracts to enhance insulation, while in warm conditions, it expands to increase breathability, eliminating the need for bulky fabrics. This technology is particularly beneficial for individuals with thermoregulation disorders such as multiple sclerosis, diabetes, or spinal cord injuries, who struggle to maintain a stable body temperature. By automatically adjusting insulation levels, these garments provide personalized thermal management, improving patient mobility and comfort without relying on external heating or cooling devices.^[^ ^156^ ^]^ Beyond medical applications, military personnel, extreme sports athletes, and outdoor workers can benefit from climate‐responsive apparel, ensuring optimal performance and protection in harsh environments.

#### 5.3.2 Smart Compression Wearables for Sports and Rehabilitation

Beyond thermal regulation, magneto‐responsive SMPs in compression garments provide programmable muscle support and injury prevention. Unlike conventional compression wear, which offers static pressure levels, these 4D‐printed textiles can dynamically adjust their fit based on muscle activity, fatigue, or injury status. This feature is particularly valuable for athletes and rehabilitation patients, where targeted muscle compression can enhance circulation, reduce swelling, and accelerate recovery.^[^ ^156^, ^157^, ^158^ ^]^ For instance, a self‐tightening knee brace can provide variable compression throughout the day, loosening for comfort and tightening when additional joint stability is required. Similarly, smart compression sleeves could be programmed to increase or decrease pressure in response to real‐time muscle fatigue detection, reducing the risk of overuse injuries. By integrating Fe_3_O_4_‐enhanced SMP fibers, these wearables provide a dynamic, user‐adaptive solution that enhances performance, injury prevention, and rehabilitation without requiring manual adjustments.

#### 5.3.3 Wearable Biosensors for Health Monitoring

The integration of magneto‐responsive SMPs with biosensing technology allows for the development of next‐generation health monitoring textiles capable of responding dynamically to physiological changes. These smart garments can be programmed to adjust their fit, compression, or stiffness based on biometric readings such as body temperature, hydration levels, blood flow, or muscle activity.^[^ ^159^ ^]^ For example, in patients with circulatory disorders, the fabric could automatically increase compression when reduced blood flow is detected, improving vascular support without requiring external intervention. Similarly, real‐time adjustments in clothing fit can provide early indicators of dehydration or overheating, making these materials particularly useful in high‐performance sports, space travel, and long‐duration medical monitoring. By combining biocompatible Fe_3_O_4_‐enhanced SMP fibers with wearable sensors, this technology paves the way for customized, self‐regulating medical wearables that bridge the gap between functional fashion and health monitoring. A summary of potential applications of 4D‑printed magnetically responsive materials, along with their functionality, advantages, and challenges, is provided in **Table** **3**.

**Table 3:** Summary of potential applications of 4D‐printed magnetically responsive materials, highlighting their functionality, advantages, and challenges.

Applications	Functionality	Importance and Advantages	Disadvantages and Challenges
Bone Tissue Engineering	Shape recovery for fracture healing	Non‐invasive, promotes regeneration, eliminates secondary surgeries, customizable, and biodegradable.	Requires precise control of shape recovery to avoid incorrect implantation; the degradation rate must match the healing speed.
Cancer Therapy	Magnetic hyperthermia for tumor treatment	Selective heating minimizes healthy tissue damage, enables combination with drug delivery, non‐invasive	Requires careful temperature control to prevent overheating; potential limitations in deep‐seated tumors due to the limited penetration depth of magnetic fields.
Smart Drug Delivery	Magnetically triggered, on‐demand release.	Improves dosage control, enhances efficacy, reduces overdosing, and side effects	Possible material fatigue over repeated activations; nanoparticle distribution must be uniform for controlled release
Wearable Medical Devices	Dynamic, self‐adjusting supports	Enhances rehabilitation, improves patient comfort, and reduces the need for multiple devices	Limited by material durability over long‐term use; requires optimized energy‐efficient magnetic actuation
Minimally Invasive Surgical Tools & Stents	Magneto‐responsive SMP stents can be inserted in a compact form and expanded remotely.	Ensures precise placement, improves patient recovery, and reduces long‐term complications from rigid stents.	Risk of incomplete expansion, precise control requirements, and potential long‐term biocompatibility concerns.
Smart Fluidic Systems for Precision Control	4D‐printed SMP‐based valves dynamically regulate liquid and gas flow through remote magnetic control.	Improves efficiency, reduces mechanical complexity, and enables real‐time reconfiguration in fluidic systems.	Limited response time, challenges in achieving fine‐tuned control, and stability issues over extended use.
Deployable Structures	Compact, lightweight structures for engineering applications expand upon magnetic stimulation.	Reduces payload weight, extends mission longevity, and eliminates mechanical failures in space applications.	Material fatigue over multiple activation cycles, complex integration into large‐scale systems, and high production costs.
Bio‐Inspired Actuators	Soft robotics and actuators dynamically change shape and recover in response to external magnetic fields.	Enables contactless shape reconfiguration, enhances adaptability, and increases robotic efficiency and safety.	Limited control resolution, energy loss during actuation, and need for improved material longevity.
Self‐Healing Materials for Enhanced Durability	Magnetic heating triggers localized self‐healing in high‐performance materials, restoring structural integrity.	Extends material lifespan, reduces maintenance costs, and enhances reliability in harsh environments.	Requires optimized formulations to ensure healing effectiveness and potential long‐term degradation concerns.
Adaptive Clothing for Climate Control & Thermoregulation	4D‐printed fabrics adjust insulation levels via magnetically controlled expansion and contraction.	Eliminates the need for bulky clothing, enhances thermal regulation, and improves mobility in extreme conditions.	Material fatigue over repeated expansions, durability concerns, and the need for precise tuning of magnetic response.
Smart Compression Wearables for Sports and Rehabilitation	Compression wear dynamically tightens or loosens based on muscle fatigue or injury, enhancing circulation and recovery.	Prevents overuse injuries, improves athletic performance, and provides adaptive muscle support.	Challenges in integration with wearable electronics, potential discomfort, and the need for long‐term stability testing.
Wearable Biosensors for Health Monitoring	Magneto‐responsive SMP biosensors adjust fit and stiffness based on real‐time biometric readings.	Enables early detection of health risks, improves circulatory support, and enhances long‐duration medical monitoring.	Sensitivity of biosensors to environmental factors, challenges in achieving seamless integration, and material durability issues.

## 6 Conclusion

Magnetically activated SMP nanocomposites have emerged as a transformative class of materials in the rapidly evolving landscape of 4D printing. This review has provided a comprehensive analysis of their fundamental mechanisms, fabrication techniques, and diverse applications, emphasizing their potential in biomedicine, soft robotics, and adaptive structures. By integrating magnetic fillers into SMP matrices, these composites enable remote, non‐invasive, programmable actuation, overcoming many limitations of conventional shape memory materials.

The discussion has highlighted the dominant 4D printing approaches, material extrusion and vat photopolymerization, as the primary fabrication methods for magneto‐responsive SMPs, each offering unique advantages in precision, scalability, and material versatility. While significant progress has been made in enhancing the mechanical, thermal, and actuation properties of these composites, challenges remain in optimizing filler dispersion, improving stability, and achieving more complex multi‐stimuli responses.

Future research should focus on refining processing techniques to enhance filler distribution, developing novel SMP formulations with tunable properties, and exploring hybrid activation strategies that combine magnetic fields with other stimuli for more sophisticated functionalities. With continued advancements in materials science and additive manufacturing, magneto‐responsive SMPs are poised to drive innovation in next‐generation reconfigurable systems. This review serves as a critical reference for researchers and engineers working at the intersection of materials engineering, smart manufacturing, and functional device development.

## Conflict of Interest

The authors declare no conflict of interest.
